# Animal acoustic communication has a conserved optimal rhythm within the neural delta range

**DOI:** 10.1371/journal.pbio.3003798

**Published:** 2026-06-09

**Authors:** Theophane Piette, Chundra Cathcart, Chiaria Barbieri, Keesha Martin Ming, Didier Grandjean, Balthasar Bickel, Eloïse Déaux, Anne-Lise Giraud

**Affiliations:** 1 Department of Basic Neurosciences, Faculty of Medicine, University of Geneva, Geneva, Switzerland; 2 Institute for the Interdisciplinary Study of Language Evolution (ISLE), University of Zurich, Zurich, Switzerland; 3 Department of Evolutionary Biology and Environmental Studies, University of Zurich, Zurich, Switzerland; 4 Department of Life and Environmental Sciences, University of Cagliari, Cagliari, Italy; 5 Swiss Center for Affective Sciences, University of Geneva, Geneva, Switzerland; 6 Université Paris Cité, Institut Pasteur, AP-HP, Inserm, Fondation Pour l’Audition, Institut de l’Audition, IHU reConnect, Paris, France; University of California Davis, UNITED STATES OF AMERICA

## Abstract

Acoustic communication is crucial for survival across the animal kingdom, with acoustic signals being shaped by the interaction of producer and receiver selective pressures. While spectral features’ variation reflects species-specific selection, the evolutionary history of acoustic communication rhythms, i.e., the rhythmic modulations of acoustic signals, remains unknown. Using data from 98 species spanning primarily mammals and birds, with additional representation from amphibians, reptiles, fishes, and insects, we investigate the origins of acoustic communication rhythms, notably whether they are shaped by the producer’s anatomical characteristics, environmental constraints, or social complexity. Regression models which controlled for phylogenetic relatedness did not support an influence of these species-specific selective forces; instead, explicit phylogenetic models of trait evolution showed that most species’ rhythms are conserved around an evolutionary optimum of 2.7 Hz that falls within the neural delta range (1–4 Hz) and predates mammalian divergence. Given the known conserved brain oscillations across species and delta involvement in active sensing, we propose that, unlike spectral features, acoustic rhythm could be governed by a universal neural mechanism facilitating effective intra and interspecific communication via a shared channel that has persisted through evolutionary times.

## Abstract 2

Animal vocalizations are diverse, yet their temporal structure is poorly understood. By analyzing acoustic rhythms across nearly 100 species, this study identifies a conserved slow tempo centered around 2.7 Hz, within the delta range, suggesting a constraint rooted in conserved neural mechanisms of auditory perception.

## Introduction

Acoustic signals enable effective and instantaneous communication between individuals, even over considerable distances. For these signals to be functional, their structure must convey adaptive information. The importance of spectral features, such as frequency content and pitch, in achieving this has been well established [1–5]. However, acoustic signals are structured not only spectrally but also temporally. Rhythms, low-pass temporal modulations of the acoustic content, also known as the sound envelope, play equally important communicative roles. This is particularly well exemplified by human speech, where speech slow modulations are sufficient for comprehension [6,7]. In most languages, syllable production rate ranges between 4 and 9 syllables per second [8]. This range corresponds to theta neural oscillations, which flexibly adapt to the syllabic rhythm during speech perception, such that modifying this flow impedes this process and reduces speech intelligibility. Rhythmic patterns allow the identification of syllables, but also words, and sentences. At a slower timescale, delta oscillation (1–4 Hz) aligns with prosodic features, and can help convey meaning, intonation, and emotional state [9,10]. In animal calls, such temporal features are no less important, for instance, in vocal recognition [11], mating behavior [12], and predator avoidance [13].

Thus, the temporal patterning of acoustic sequences carries significant communicative functions and is not specific to speech. However, questions remain regarding the factors that influence the evolution of rhythm across the animal kingdom. In this study, we explore acoustic communication rhythms across diverse animal clades, test leading hypotheses on the evolution of signal structure, and model the most plausible evolutionary scenarios. Taking a macroevolutionary perspective, we examine whether large-scale patterns of rhythm variation across species can be explained by shared evolutionary constraints. We consider four major selective forces that may drive the evolution of rhythm. First, the presence of theta rhythms in the vocal productions and mouth movements of non-human primates suggests that such rhythms may originate from the natural oscillatory movements of the articulators, inherited directly from mastication [14–16]. Second, in vocalizing animals, morphological and physiological factors, such as breathing rate, heart rate, or metabolism, may constrain rhythmic range in a manner analogous to constraints on spectral features [17]. Third, for acoustic signals to be effective, they must reach receivers despite environmental constraints, which could shape not only spectral but also temporal features of communication [18,19]. Additionally, the social complexity hypothesis proposes a positive relationship between the complexity of an animal’s social environment and the complexity of its vocal repertoire [20]. Since rhythm determines the rate at which information can be transmitted, social complexity could also positively influence acoustic tempo, as species with more complex social systems may need to convey more information within a given time frame. Finally, it is possible that rhythm evolution is not shaped by species-specific selective pressures, but rather by phylogenetic processes of conservation and diversification that are either shared across, or vary between, lineages.

To test these different hypotheses, we quantified rhythm in acoustic sequences primarily from birds and mammals, with additional, more limited data from amphibians, insects, reptiles, and fish. Using Bayesian multilevel models for phylogenetic regression [21], we controlled for phylogenetic relationships and evaluated, in accordance with the previously stated hypotheses, whether weight (as a proxy for breathing rate, heart rate, and metabolism), mastication status, sociality level or ecological characteristics could account for differences in rhythm. Finally, we compared phylogenetic models capturing contrasting evolutionary scenarios, asking whether rhythm shows signatures of convergence toward a shared optimum, as expected under an Ornstein–Uhlenbeck (OU) evolutionary process, or whether it has diversified through unconstrained, Brownian-like divergence across lineages.

### Rhythm computation

**Fig 1:**
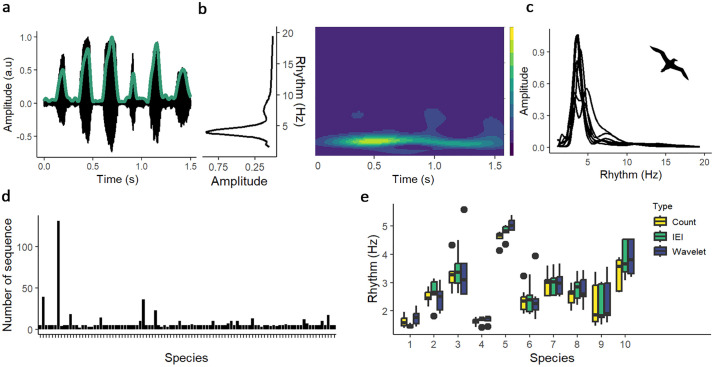
Methodology. **a)** Oscillogram of one acoustic sequence of polar skua call, green line shows the computed signal envelope. **b)** Power spectrum and time-frequency representation of the envelope of the previous sequence. **c)** Power spectra of the envelopes of all polar skua acoustic sequences. **d)** Number of sequences per species (ordered by alphabetical order). **e)** Rhythm computation using sequences of 10 randomly selected species, employing, from left to right, number of elements per second (Count), inter-element intervals (IEI), and wavelet method (Wavelet). The data underlying this Figure can be found in https://zenodo.org/records/19816728.

To explore the evolution of communication rhythm in animals, we analyzed acoustic sequences from 98 species (58 birds, 28 mammals, 4 amphibians, 4 insects, 1 reptile, and 1 fish). We calculated rhythm by analyzing the low-pass variations in the signal amplitude, allowing broad applicability across species (Fig 1a–1d). Validation against conventional methods showed consistent results (Fig 1e: *F*_2.171_ = 0.33, *p* = 0.72), confirming the robustness of our approach. Control of the impact of signal-to-noise ratio (SNR) and sequence length revealed no significant relationship with rhythm (S1a and S1b Fig). Additional investigation of the allometric relationship between animal weight and the dominant frequency of acoustic signals (S2 Fig), and of the effect of context on rhythm (S4a Fig) further confirmed the validity of our acoustic database.

### Species-specific selective pressure

**Fig 2:**
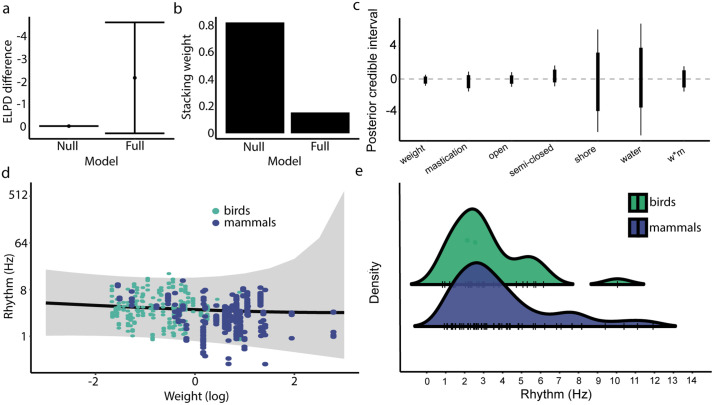
Bayesian multilevel model. **a)** Leave-one-out expected log pointwise density difference (ELPD) between the null and the full distributional (scale-location) models. **b)** Stacking weight of the models. **c)** Posterior credible intervals (95% and 85%) of the full distributional model (W*M = Weight*Mastication). **d)** Rhythm plotted as a function of log-transformed weight with predicted slopes from the full distributional model and their standard error. **e)** Distribution of raw median rhythm (in Hz) across birds and mammals. Each vertical black line represents the median rhythm of a single species. The data underlying this Figure can be found in https://zenodo.org/records/19816728.

To investigate which factors influence the evolution of rhythm, we fitted two phylogenetic regressions in a Bayesian multilevel framework. A full model investigating the impact of weight, mastication status, and living environment while controlling for phylogenetic relatedness, and a null model controlling for phylogenetic relatedness only. Because insects, fish and anurans are underrepresented in our dataset, we restricted the comparative evolutionary analysis to birds and mammals to ensure robust phylogenetic inference. We extended the analyses to the full dataset in the supplementary materials as an exploratory approach, providing an indication of potential generalization. We compared models via their leave-one-out expected log pointwise density (ELPD) and stacking weight. Due to heteroskedasticity, distributional (scale-location) models better fit the data, leveraging over 90% of the stacking weights (S3 Fig). Including the predictors did not improve predictive performance (Fig 2a), and the null model leveraged the highest stacking weight (Fig 2b). The posterior distribution of the regression coefficients of the full model revealed that none of our predictors had any decisive effect on rhythm, given that their 95% credible intervals (CI) all contained zero. Taken together, these results indicate that including these predictors does not add decisive explanatory value to phylogenetic history. The supplementary analyses including all species yielded comparable results, suggesting that this pattern may generalize to more distantly related taxa (S4 Fig). As vocal complexity, our proxy for social complexity in vocalizing species, and beak morphology in birds were not available for all species, these parameters could not be included in the main phylogenetic model. We therefore conducted separate linear mixed model analyses, regressing rhythm on each factor, with phylogenetic class and order as random effects to account for evolutionary influence. These analyses revealed no significant relationship between rhythm and either vocal complexity (S5d Fig: *t* = −0.75, *p* = 0.46) or beak morphology (S5e–S5g Fig: *t* = −1.4, 0.98, 0.35; *p* = 0.17, 0.33, 0.73).

### Phylogenetic history of rhythm

**Fig 3:**
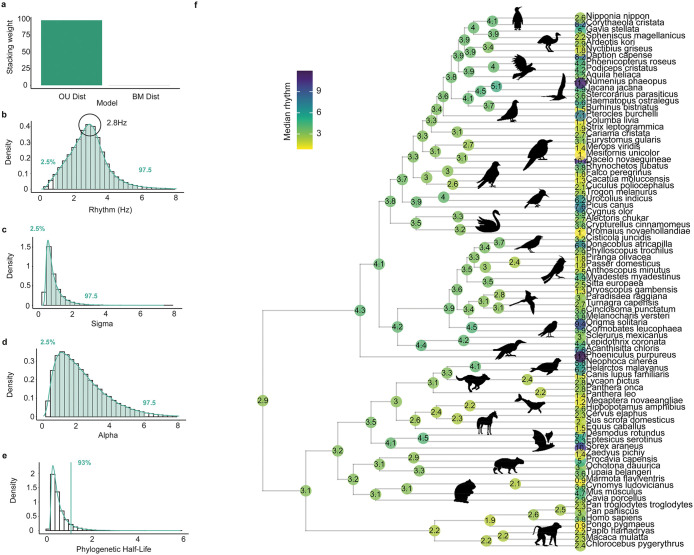
Phylogenetic modeling. **a)** Stacking weight of the OU and BM distributional models. **b)** Posterior distribution of rhythm in the OU distributional model. **c)** Posterior distribution of sigma, the scale of the drift process. **d)** Posterior distribution of alpha, the strength of selection. **e)** Posterior distribution of the phylogenetic half-life. **f)** Visualization of reconstructed median posterior values using a maximum clade credibility (MCC) tree. The data underlying this Figure can be found in https://zenodo.org/records/19816728.

Visual inspection of the median rhythm for our tested species shows that in every class, acoustic rhythm spans mainly the lower rates <5 Hz (Fig 2d). To understand if the produced rhythms have randomly evolved within this range, favoring species-specific rhythm, or have been maintained around an optimum value, we fitted models of the evolution of vocal rhythm under Brownian Motion (BM) and OU processes [10], representing each evolutionary scenario, respectively. Comparisons of ELPD values and model stackings show that the OU model best fits the data (Fig 3a). The median posterior estimate of rhythm is 2.7 Hz, with 95% CI of [0.45, 4.99] Hz (Fig 3b). This represents both the optimum to which the OU process reverts over time and the likely state at the root of the phylogeny. The posterior distribution of sigma represents the stochastic volatility (Fig 3c) and has a median of 0.68 and a 95% CI of [0.35,2.37]. The posterior distribution of alpha represents the strength of attraction (Fig 3d) and has a median of 1.95 and a 95% CI of [0.42,6.01]. The proportion of posterior half-life estimates lower than 1 (height of the tree) is 95% (Fig 3e), supporting strong selection with fast reversals to the optimum. Consistent with this, rhythm values close to the optimum are also reconstructed for most interior nodes of the phylogeny (Fig 3f). In summary, the model suggests that there is a phylogeny-wide evolutionary pressure towards an optimal rhythm, to which species that would deviate quickly revert to. This result is further supported by models fitted separately for mammals and birds, where the OU model consistently received the highest stacking weight (birds: wOU = 1, mammals: wOU = 0.925) and showed a similar pattern of slow rhythm conservation (S6 Fig). In addition, a supplementary model that included anuran, insect, and fish species yielded comparable outcomes (S7 Fig), suggesting that the conservation of slow acoustic rhythm may have an even older evolutionary origin.

### Discussion

In this exploratory study, we report an optimum rhythm for acoustic communication in birds and mammals with credible generalization to additional clades (insects, anurans, fish), challenging the idea that it would be shaped by biomechanical constraints. A biomechanical influence would predict distinct rhythms between masticating and non-masticating species, along with a strong negative allometric relationship between rhythm and weight in masticating species. Instead, our analyses show that rhythm evolution cannot be explained by weight, environmental pressures, or social complexity (Figs 2c and S5d). This surprising absence of effect indicates that biomechanical constraints exert only a limited influence on acoustic communication rhythms at the macroevolutionary scale. By contrast, control analyses on the signals’ dominant frequency (S2 Fig) reconfirmed that spectral characteristics primarily depend on the specifics of the production organ, which chiefly vary with the animal’s weight [11]. Given the minimal contribution of physical characteristics, the lack of explanatory power from environmental and social variables, and the diversity of auditory structures and production mechanisms across taxa, it is unlikely that this shared rhythm results from similarities in anatomy alone.

A key insight comes from comparing potential evolutionary scenarios for rhythm. The phylogenetic analyses clearly favored an OU model over a BM model, indicating strong stabilizing selection rather than unconstrained divergence (Fig 3). This result strengthens the view that species-specific factors, although capable of introducing local variation, do not account for the large-scale conservation of rhythm across distant taxa. With regard to its evolutionary origins, the presence of a similar rhythm across birds and mammals suggests that it was already present in their last common ancestor roughly 340 million years ago (Fig 3). Although insects, amphibians, and fishes are represented by fewer species in our dataset, the presence of slow rhythms in these taxa may hint at an even earlier emergence (S7 Fig). While other factors such as temperature or reverberation are also known to influence communication rhythms in ectotherms, such short-term and context-specific effects are unlikely to generate the macroevolutionary pattern observed here. These findings prompt the consideration of alternative explanations, potentially rooted in neural rather than anatomical, environmental or social factors. As neural mechanisms are essential for survival, they tend to be highly conserved across species and may offer a more plausible basis for a widespread optimal rhythm in communication. Although the size of neurons varies across species, their conductance is kept relatively constant through compensatory variations in ion channel density [22]. This results in conserved time constants for neural responses, including oscillatory phenomena [23].

The conserved rhythm found here around 2.8 Hz (95% interval 0.45–4.99 Hz) best matches delta brain oscillations (1–4 Hz). Interestingly, delta-range oscillations have been described across a wide range of taxa, including mammals, birds, reptiles and insects. In mammals, conserved oscillatory patterns have been reported from humans, primates, carnivores and rodents, with delta among the most stable across species [23]. In reptiles, such as turtles, delta activity dominates brain rhythms and supports ancient functions such as selective attention [24]. Even in birds and insects, delta oscillations have been observed during sleep or quiescent states, indicating that slow oscillatory activity is widespread throughout the animal realm [25]. Delta oscillations support slow perceptual integration and are engaged during active sensing behaviors such as sniffing or blinking [26], suggesting that they provide a fundamental temporal framework through which organisms sample and organize incoming sensory information. Because acoustic signals contain information distributed across both slow and fast temporal scales, as shown in our control analyses on context (S4 Fig), a conserved slow rhythm implies the need for a complementary faster mechanism capable of resolving finer temporal details. Within this neural framework, slow and fast timescales likely serve complementary functions. Delta oscillations would provide a long integration window that supports the identification of acoustic structure, while faster processes (most likely low-gamma) would enable rapid detection and fine temporal discrimination. This division of labor echoes the Fourier uncertainty principle, which states that long temporal windows allow precise frequency resolution at the expense of temporal precision, whereas short windows offer the opposite trade-off [27]. In this view, delta rhythms provide the temporal window best suited for integrating the broad acoustic patterns that carry communicative meaning, while faster oscillations handle local, transient features. Because the present study relies on large-scale comparative acoustic data, it naturally captures the slow integrative timescale rather than the fast detection range. Understanding how these slow and fast processes interact to shape acoustic communication will require dedicated neurophysiological work in future studies.

The independence and parallel functioning of slow and fast scales acoustic processing is attested by human psychophysics findings. When time-compressed unintelligible speech is chunked and repackaged by interspersing silent gaps to restore regular syllable and word rates, speech becomes intelligible again. These results show that speech comprehension is limited by the packaging of information at slow rhythm, rather than by the absolute capacity to decode accelerated segmental (phonemic) cues [6,28]. This emphasizes the relevance of slow rhythms in maintaining acoustic signal decodability. Human speech operates at a mean rate that lies at the higher end of the median universal rhythm identified in this study, clustering around 5 Hz across all languages [8]. This reflects humans’ tendency to convey more information per unit of time compared to other species. Dogs, for instance, despite millennia of domestication and a demonstrated ability to understand many aspects of human language [29], process speech at a slower rhythm, typically between 1 and 3 Hz, than the human average of 5 Hz [30]. While they do not track the syllabic rhythm of speech, dogs tend to process it more globally, at the word rate, which aligns with their own vocal production rhythm (e.g., bark rate). These comparative findings highlight species-specific variations in preferred communication rhythms, while also underscoring a broader pattern: a conserved and effective temporal resolution for communication that enables interspecies sensitivity to acoustic signals. In sum, the conserved production rhythm identified here aligns closely with an equally conserved neural rhythm that facilitates the assignment of meaning to acoustic input, ultimately supporting an effective and evolutionarily robust communicative system.

In conclusion, the maintenance throughout evolution of a common slow rhythm for acoustic signal production and perception de facto results in a common communication channel across coexisting species, offering possibilities for interspecies signaling (e.g., a common danger) and/or eavesdropping, and thus conferring evolutionary advantages. Spectral (frequency) and temporal (rhythm) features, both key to effective communication, seem to have followed different trajectories. While spectral traits diversify with the production and hearing organ anatomy, acoustic rhythms might be shaped by basic neural factors essential for survival that have led to a slow conserved optimum. Whether these neural factors were maintained because they amounted to an optimal communication design, or whether this design arose from recycling vital and highly conserved neural traits (neural rhythmicity via maintained neuronal conductance) remains to be elucidated. A further interesting avenue will be to probe for an optimum that combines fast and slow timescales in acoustic communication, alongside testing the delta wave hypothesis by assessing whether interspecific variation in rhythm is accompanied by corresponding variation in neural timescales.

## Materials and methods

### Vocal sequences

To perform an extensive phylogenetic comparison on a balanced phylogeny, we collected acoustic and biological data for at least one species per infra-order of tetrapods, when data were available, as well as a few species of insects and fishes, to obtain a good representation of rhythm throughout the phylogeny. Acoustic sequences were gathered from public and private databases (Fonozoo, Cisro, Berlin Museum fur Naturkunde and Xeno-canto), online videos platform (Youtube, Dailymotion), and from different research groups that kindly shared audio files.

Following a cross-species literature survey [31–34], we defined a sequence as a recording of an acoustic display produced by a single individual, containing more than two calls separated by less than two seconds of silence. For computational purposes, we only retained sequences lasting more than one second.

Data collection then followed a strict and standardized protocol. For each order, we first screened public databases and ranked species by the number of available sequences. We then selected the first species fulfilling the following criteria: at least five clean sequences (non-noisy, with a single individual vocalizing at a time) originating from different individuals. Once such a species was identified, we proceeded to the next infraorder. When no species meeting these criteria was available, we extended the search to private databases and directly contacted researchers. If no data could be obtained, that infraorder was excluded.

Only recordings with minimal background noise and a clearly identifiable focal individual were retained; any containing rhythmic background noise, overlapping individuals, or ambiguous sound sources were excluded. This careful filtering minimized the risk of confounding effects and ensured that the extracted acoustic envelopes reliably reflected the focal signal across different sources.

The final dataset comprised 98 species: 58 birds, 28 mammals, 4 amphibians, 4 insects, 1 reptile, and 1 fish. Species with fewer than five sequences were included only when they represented the sole available species for their infraorder.

### Weight

Weight presents an allometric relationship with morphological features and physiological processes involved in acoustic productions across various species. Thus, we collected biological data on the mean weight for each species, to serve as a proxy for heart rate, breathing rate, and metabolism. This involved calculating the average weight by considering both the minimum and maximum weights recorded for each species, irrespective of sex. These data were primarily obtained from the handbook of mammals of the world [35], and the handbook of birds of the world [36]. When data were unavailable from these sources, we looked for reference articles.

### Beak size

Just as masticatory abilities may have influenced rhythm in mammals, a similar proposition could be made regarding beak morphology in birds. Unlike other animals, the morphological traits of a bird’s beak do not consistently adhere to an allometric relationship with its weight [37]. Thus we collected information on beak length, width and depth of our species. As these measures were not available for all species, and only applicable to birds, we could not include them in the phylogenetic model. We therefore built an additional linear mixed model investigating the variation of rhythm, including phylogenetic class and order as random effects to account for evolutionary influence, and beak length, width, depth, and their interactions as fixed effects. Data are available in the study github.

### Living environment

As environmental conditions can impact vocal communication [38], we also collected data on the typical habitat of each species. We used a five level categorical classification, with habitats being either classified as closed (defined as habitats with heavy tree coverage), semi-closed (defined as habitats with light three coverage or human cities), open (defined as fully open habitat with no three coverage or obstacle), shore (for species living near a significant amount of water such as lakes, rivers or seas) or water (for species living below the water surface). These data were primarily obtained from the handbook of mammals of the world, and the handbook of birds of the world. When data were unavailable from these sources, we looked for reference articles. Data and linked references are available in the study github.

### Mastication status

As some have proposed that rhythmic communication in vocalizing animals may be linked to mastication regime [14–16], we also classified each species according to their mastication status (yes or no).

### Social complexity

As social complexity increases, individuals may need to communicate more information in a given time, and therefore speed up their communication. As communication signals have been linked to social complexity [20], we also gathered species vocal repertoire complexity (number of distinct calls in the species vocal repertoire) when this information was available. As this measure was not available for all species, we could not include it in the phylogenetic model. We therefore built an additional linear mixed model investigating the variation of rhythm, including phylogenetic class and order as random effects to account for evolutionary influence, and vocal complexity as fixed effect. These data were primarily obtained from the handbook of birds of the world, and reference articles. Data and linked references are available in the study github.

### Rhythm analyses

To quantify rhythm in these acoustic sequences, we decided to adapt the method developed by Tilsen and colleagues to compute rhythm in human speech production [39]. This method uses the signal amplitude to automatically compute the rhythmic component of a sequence, without making any assumption on the components’ size, and is thus widely applicable across all species regardless of variations in unit size or spectral characteristics. First, we denoised the sequences using a first-order Butterworth filter, with a bandpass filter between a minimal frequency (minF), defined as 200 Hz below the minimum frequency of the animal call, and a maximum frequency (maxF), defined as 200 Hz above the maximum frequency of the animal acoustic signal obtained from reference articles. When this information was not available, we applied a large range filter with a 100 Hz minF and 10,000 Hz maxF. We then computed the normalized envelope of the denoised sequences using the Hilbert Transform. Next, we low-pass-filtered this envelope with a fourth-order Butterworth filter with a 20 Hz cut-off frequency to obtain the slow changes in acoustic energy. Before further analysis, we downsampled the resulting signal at 150 Hz for computational purposes, and then applied a continuous wavelet transform using the Morlet wavelet to obtain a time-frequency representation of the amplitude envelope. We replaced the Fourier transform with a wavelet transform, to allow for more flexibility with regards to the variation in sequence length present in our dataset. We finally analyzed that representation’s power spectrum to extract the five frequency peaks of highest amplitude in the power spectrum and used the time-frequency representation to select the main rhythmic component conserved across the entire sequence.

To assess the validity of the proposed methodology, we conducted a comparative analysis between the calculated vocal rates of a subset comprising 10% of our database and those derived from two widely accepted conventional approaches: (1) by counting the number of elements per second (Count) and (2) by computing the inter-element interval (IEI). The three methods gave sensibly similar results (Fig 1d: *F*_2.171_ = 0.33, *p* = 0.72), hence validating our rhythm quantification method.

### Signal-to-noise ratio (SNR) and length

As further control analyses, we quantified recording durations in seconds and SNR in decibels. To control for the effect of both factors and their interaction on rhythm, we build a linear mixed model investigating the variation of rhythm including group and order as random effects, and SNR, length and their interaction as fixed effects.

### *Dominant frequency analyse*s

To determine dominant frequencies, which unlike the fundamental frequency are measurable in all types of communicative signals [40], we isolated the first acoustic unit in each denoised sequence. We then applied a single discrete Fourier transform to compute the power spectrum of these units, and extract the peak of highest amplitude. The obtained results were also visually controlled in Praat, to make sure that the extracted dominant frequency matched the acoustic energy present in the unit. If the first unit had poor SNR leading to inaccurate computation of the dominant frequency, we selected the next unit in the sequence.

### Context effect control

While the existing literature highlights the importance of context and its correlated arousal levels on acoustic signal rate [1], for most species we were not able to obtain these data. Nevertheless, whenever possible we selected recordings of different call types for each species. Further, we performed separated analysis of variance (ANOVA) to control for the effect of call type on rhythm in three species: one avian and two mammalian, from which we could obtain different call types, including contact calls, alarm calls, songs, agonistic and antagonistic vocal displays.

### Phylogenetic tree sample reconstructed from genetic sequences

To test our hypotheses of interest we used phylogenetic comparative methods (PCM), a broad family of methodological tools for characterizing and controlling for the evolutionary dynamics thought to give rise to the data under study. PCMs require a representation of the relatedness of the taxa under study in the form of a phylogenetic tree sample. To represent the tree topology of the species we performed a phylogenetic analysis based on comparable genetic sequences, using a Bayesian framework to infer a posterior tree sample. We first matched each species in the sample with their closest genetic proxies in GenBank [41], and extracted mitochondrial DNA for the corresponding species. For 54 of the 98 species, matching mitochondrial genomes were available from literature and deposited in GenBank. For the 44 remaining species we chose proxies from another closely related species. We took a species within the same genus when possible; if this was not available, we chose species within the family of the target species, after confirming that no more than one species per family was included in the original list. Only for four target species—*Correlophus ciliates* (Squamata), *Galbula ruficauda* (Aves), *Leptosomus discolor* (Aves), *Phaethon rubricauda* (Aves) —we did not find proxies within the family and we had to find a proxy within the order. To choose the best mtDNA proxy with those deposited in GenBank, we considered completeness of the available mitochondrial sequences and comparable average size, weight, and environment of the target species. Maximum missing data is 100 base pairs, for an average size of 16706 base pairs. MtDNA genomes were aligned with MAFFT software [42] and standard settings. The alignment was manually screened in BioEdit (version 7.2, https://thalljiscience.github.io/) for spotting irregularities and potential outlier sequences. Sequences were then cut to keep only the coding region, which is more conserved across species, using the *Homo sapiens* sequence as a reference. The final alignment consisted of 21860 base pairs, which include large INDELs sections to accommodate alignment between the most divergent species (e.g., *Apis mellifera*).

We used BEAST2 to generate the trees, running 10′000′000 iterations of Markov chain Monte Carlo (MCMC) with a thinning interval of 1,000. We used the following settings to approximate the broad evolutionary range of the species considered: assuming an HKY substitution model, a strict clock (Uniform rates across branches), and a Birth-Death tree prior with a Yule birth rate. This resulted in 10′000 trees, of which we use 50 for phylogenetic comparative analyses.

### Bayesian multilevel models for phylogenetic regression

To assess the impact of several predictors of interest on different properties of variation in vocal rhythm and dominant frequency, we used phylogenetic regression modeling, a comparative method that assesses the effect of predictors on a response while controlling for the phylogenetic relatedness of the taxa. Due to heterogeneity in the number of datapoints and individuals in each species, we employed both non-distributional regression models, which model the mean of the response variable as a function of predictors, and distributional (scale-location) regression models, which model both the mean and standard deviation of the response variable as a function of predictors. We control for species-level idiosyncrasies in both the median and (in some cases) standard deviation of rhythm via phylogenetic random intercepts and slopes (phylogenetic random effects are similar to the standard random effects used in hierarchical regression modeling, but are generated by a Gaussian Process with a covariance kernel that is a function of the phylogenetic patristic distances between species under study rather than independently and identically distributed with diagonal variance).

We fitted four phylogenetic regression models using brms [21] for each response variable (vocal rhythm, dominant frequency), resulting in eight models. Due to the constraints of bayesian multilevel modeling, particularly its sensitivity to missing data across hierarchical levels, we restricted the main model to predictors available for the majority of species. Specifically, we included average species weight (log-transformed, centered, and standardized) [43], mastication status, and species’ living environment. We also modeled the interaction between mastication status and weight. Additional predictors that were only available for a limited subset of species were analyzed separately in independent linear mixed models to avoid excessive data imputation or loss of statistical power.

Our distributional models have the following basic generative process (below, $\alpha^{\mu }+\beta X_{i\;}$ is shorthand for all model predictors, including fixed and random effects):


$$\begin{array}{c} y_{i}\;\sim \;LogNormal(\mu_{i},\;\sigma_{i}) \end{array}$$

$$\begin{array}{c} \mu_{i}\;=\;\alpha^{\mu }\;+\;\beta^{\mu }X_{i\;} \end{array}$$

$$\begin{array}{c} \sigma_{i}\;=\;exp(\alpha^{\sigma }+\;\beta^{\sigma }X_{i}) \end{array}$$

Non-distributional models have the following structure:


$$\begin{array}{c} y_{i}\;\sim \;LogNormal(\mu_{i},\;\sigma ) \end{array}$$

$$\begin{array}{c} \mu_{i}\;=\;\alpha^{\mu }\;+\;\beta^{\mu }X_{i\;} \end{array}$$

We employ the default priors of brms.

The first of the four models was a full distributional one that modeled both the expected median and variance of the response variable as a function of these predictors, while controlling for species-level idiosyncrasies in both the median and variance of rhythm via phylogenetic random intercepts and slopes. The second was a full non-distributional model that treated only the expected median rhythm as a function of the predictor variables as well as phylogenetic random intercepts and slopes. The third of these was a null distributional model that included only phylogenetic random intercepts and slopes for the expected median and variance. The final model was a null non-distributional that included only phylogenetic random intercepts and slopes for mean rhythm. We ran each of these models for 4,000 iterations of the no U-turn sampler over 4 chains with a log-normal link function and discarded the first half of samples, aggregating posterior samples across the retained sampled trees.

Models in brms have the following formulae:

Full, distributional


$$\begin{array}{c} \text{bf}(\text{frequency }\sim \text{ weight}*\text{mastication }+\text{ environment }+\;(\text{1}\;+\text{ weight}*\text{mastication }+\text{ environment }|\text{ gr }(\text{taxon},\text{ cov }=\text{ phylo}.\text{cov})) \end{array}$$

$$\begin{array}{c} \text{bf}(\text{sigma }\sim \text{ weight}*\text{mastication }+\text{ environment }+\;(\text{1}\;+\text{ weight}*\text{mastication }+\text{ environment }|\text{ gr}(\text{taxon},\text{ cov }=\text{ phylo}.\text{cov}))) \end{array}$$

Full, non-distributional


$$\begin{array}{c} \text{bf}(\text{frequency }\sim \text{ weight}*\text{mastication }+\text{ environment }+\;(\text{1}\;+\text{ weight}*\text{mastication }+\text{ environment }|\text{ gr}(\text{taxon},\text{ cov }=\text{ phylo}.\text{cov}))) \end{array}$$

Null, distributional


$$\begin{array}{c} \text{bf}(\text{frequency }\sim \;(\text{1}\;|\text{ gr}(\text{taxon},\text{ cov }=\text{ phylo}.\text{cov})),\text{ sigma }\sim \;(\text{1}\;|\text{ gr}(\text{taxon},\text{ cov }=\text{ phylo}.\text{cov}))) \end{array}$$

Null non-distributional


$$\begin{array}{c} \text{bf}(\text{frequency }\sim \;(\text{1}\;|\text{ gr}(\text{taxon},\text{ cov }=\text{ phylo}.\text{cov}))) \end{array}$$

We compared fitted models via their leave-one-out ELPD values [44] and stacking [45], which average predictive distributions of different models to generate weights representing their relative predictive power. We used the function loo_compare to measure differences in ELPD across models. Finally, we inspected posterior distributions of regression coefficients of the full distributional model to assess the effects of predictors of interest.

### Evolutionary dynamics of vocal rhythm

We further investigate the properties of rhythm across species using two Gaussian Process models of continuous trait evolution, asking specifically whether the evolution of vocal rhythm is characterized by a random process of drift (characterized by BM) or whether selective forces draw rhythm values toward an optimal value over time (a mean-reverting scenario characterized by an OU process). Under BM, the displacement of a continuous trait at time *s* has a variance proportional to the amount of time elapsed over the course of displacement (below denoted as *t*), where σ represents the scale of the drift process:


$$\begin{array}{c} X(s)\;\sim \;Normal(X(s-t),\sigma t) \end{array}$$

Under an OU process, the displacement of a character has the following formula:


$$\begin{array}{c} dX(s)\;=\;\alpha (\theta \;-X(t))dt\;+\;\sigma dW(t) \end{array}$$

In the first component of the sum, α represents the strength of selection to the optimal value θ. The second component represents a process of BM, with σ representing the scale of drift. Thus, the OU process allows for both selective and random forces in character evolution.

An standard way to interpret α is to transform it to the phylogenetic half-life, ln 2/*α* [45]. This is interpreted as the average time for a trait to evolve halfway from an ancestral state toward a new optimum, indicating how long it will take before adaptation to a new regime is more influential than constraints from the ancestral state. If half-life values are greater than the height of the phylogeny (1 in our case, as the tree length is scaled to unit height), the process increasingly resembles BM and involves a slower adaptation speed.

As above, we employ a distributional approach, allowing species-level mean rhythm values and species-level standard deviations of rhythm values to evolve over the phylogeny according to BM or OU processes.

The distributional BM process has the following generative process:


$$\begin{array}{c} y_{i}\sim \text{LogNormal}(\mu [{\text{species}}_{i}],\sigma [{\text{species}}_{i}]) \end{array}$$

$$\begin{array}{c} \mu \sim \text{MVNormal}(\overset{\mu }{\underset{\text{0}}{\theta }},\tau^{\mu }\Psi ) \end{array}$$

$$\begin{array}{c} \sigma \sim \text{MVNormal}(\overset{\sigma }{\underset{\text{0}}{\theta }},\tau^{\sigma }\Psi ) \end{array}$$

$\theta_{\text{0}}$ represents the trait value at the root of the tree, while Ψ is a matrix of the shared history (the time between the root age of the tree and the most recent common ancestor) of each pair of nodes in the tree and τ is the positive scale of drift. Conventions are as above.

The distributional OU process has the following generative process:


$$\begin{array}{c} y_{i}\sim \text{LogNormal}(\mu [{\text{species}}_{i}],\sigma [{\text{species}}_{i}]) \end{array}$$

$$\begin{array}{c} \mu \sim \text{MVNormal}(\overset{\mu }{\underset{\text{0}}{\theta }},\tau^{\mu }\text{exp}(-\alpha^{\mu }\Delta )) \end{array}$$

$$\begin{array}{c} \sigma \sim \text{MVNormal}(\overset{\sigma }{\underset{\text{0}}{\theta }},\tau^{\sigma }\text{exp}(-\alpha^{\sigma }\Delta )) \end{array}$$

$\theta_{\text{0}}$ represents the trait value at the root of the tree, τ is the positive scale of drift, *α* is the positive strength of selection, and Δ is a matrix of pairwise cophenetic distances between species in the phylogeny, scaled to a maximum distance of 1. Conventions are as above. We place *Normal(0,1)* priors over unconstrained parameters and *Gamma(1,1)* priors over positive parameters. In addition to running these models on all species in our sample, we validate results by running models on bird and mammal species alone.

### Phylogenetic reconstruction of rhythm values

Rhythm values were reconstructed to internal nodes of the maximum clade credibility (MCC) tree of the phylogeny, using ggtree package [46] by drawing 10 draws from each of the posterior distributions inferred from the 50 different trees in the tree sample and sampling values at internal nodes of the tree from the normal distribution parameterized by the OU process, conditioned at the expected tip values (Fig 3f).

### Software

All analyses and visualization were done using Stan and R version 4.1.2 (2021-11-01) with the following packages Seewave [43], Soundgen [47], DoBy [48], Lme4 [49], MuMYn [50], brms [21], ggplot2 [51].

## Supporting information

S1 Text Additional methods, validation analyses, and supplementary results supporting the main manuscript, including phylogenetic regressions, control analyses, and robustness checks. (DOCX)

S1 Fig Signal-to-noise ratio and sequence length effect on rhythm. **a)** Rhythm (Hz) as a function of signal-to-noise ratio (SNR) showing the absence of relationship between SNR and Rhythm (*t* = −0.21, *p* = 0.84, *R*^2^ = 0.02). **b)** Rhythm (Hz) as a function of recording length showing the absence of relationship between sequence length and rhythm (*t* = −1.45, *p* = 0.15, *R*^2^ = 0.02). The data underlying this Figure can be found in https://zenodo.org/records/19816728.
(TIF)

S2 Fig Dominant frequency phylogenetic regression. **a)** Leave-one-out expected log pointwise density difference (ELPD) between the full and the null distributional models. **b)** Stacking weight of the models. **c)** Posterior credible interval (95% and 85%) of the full model. **d)** Dominant frequency plotted on a logarithmic scale as a function of log-transformed weight with predicted slopes from the full distributional model and their standard error. The data underlying this Figure can be found in https://zenodo.org/records/19816728.
(TIF)

S3 Fig Rhythm phylogenetic regression. **a)** Leave-one-out expected log pointwise density difference (ELPD) between the null distributional (“dist”) model and the others (“ndist” for “non-distributional, modeling only the mean”). **b)** Stacking weight of the models. The data underlying this Figure can be found in https://zenodo.org/records/19816728.
(TIF)

S4 Fig Bayesian multilevel model including all species. **a)** Leave-one-out expected log pointwise density difference (ELPD) between the null and the full distributional (scale-location) models. **b)** Stacking weight of the models. **c)** Posterior credible intervals (95% and 85%) of the full distributional model (W*M = Weight*Mastication). **d)** Rhythm plotted as a function of log-transformed weight with predicted slopes from the full distributional model and their standard error for masticating and non-masticating species. **e)** Distribution of raw median rhythm (in Hz) across birds and mammals. Each vertical black line represents the median rhythm of a single species. The data underlying this Figure can be found in https://zenodo.org/records/19816728.
(TIF)

S5 Fig Additional analysis on rhythm. **a)** Rhythm in sequences of different call types (1 = affiliative grunt, 2 = scream, 3 = threat grunt) in olive baboons *(Papio anubis)*. **b)** Rhythm in sequences of different call types (1 = bark, 2 = growl, 3 = howl, 4 = snarl, 5 = whine) in dogs (*Canis lupus familiaris*). **c)** Rhythm in sequences of different call types (1 = alarm call, 2 = flight call, 3 = song) in Eurasian stone-curlews (*Burhinus oedicnemus*). **d)** Rhythm plotted as a function of vocal repertoire size. **e)** Rhythm plotted as a function of beak length in birds. **f)** Rhythm plotted as a function of beak depth in birds. **g)** Rhythm plotted as a function of beak width in birds. The data underlying this Figure can be found in https://zenodo.org/records/19816728.
(TIF)

S6 Fig Independent phylogenetic modeling for birds (a–e) and mammals (f–j). a,f) Stacking weight of the OU and BM distributional models. b,g) Posterior distribution of rhythm in the OU distributional model. c,h) Posterior distribution of sigma, the scale of the drift process. d,i) Posterior distribution of alpha, the strength of selection. e) Posterior distribution of the phylogenetic half-life. e,j) Visualization of reconstructed median posterior values using a maximum clade credibility (MCC) tree. The data underlying this Figure can be found in https://zenodo.org/records/19816728.
(TIF)

S7 Fig Phylogenetic modeling including all species. **a)** Stacking weight of the OU and BM distributional models. **b)** Posterior distribution of rhythm in the OU distributional model. **c)** Posterior distribution of sigma, the scale of the drift process. **d)** Posterior distribution of alpha, the strength of selection. **e)** Posterior distribution of the phylogenetic half-life. **f)** Visualization of reconstructed median posterior values using a maximum clade credibility (MCC) tree. The data underlying this Figure can be found in https://zenodo.org/records/19816728.
(TIF)

## Abbreviations

ANOVA: analysis of variance
BM: Brownian Motion
CI: credible intervals
ELPD: expected log pointwise density
IEI: inter-element interval
MCC: maximum clade credibility
MCMC: Markov chain Monte Carlo
PCM: phylogenetic comparative methods
SNR: signal-to-noise ratio

## References

[1] Briefer EF. Vocal expression of emotions in mammals: mechanisms of production and evidence. J Zool. 2012;288:1–20.

[2] Fletcher NH. A simple frequency-scaling rule for animal communication. J Acoust Soc Am. 2004;115(5 Pt 1):2334–8. doi: 10.1121/1.1694997 15139646

[3] Fukushima M, Doyle AM, Mullarkey MP, Mishkin M, Averbeck BB. Distributed acoustic cues for caller identity in macaque vocalization. R Soc Open Sci. 2015;2(12):150432. doi: 10.1098/rsos.150432 27019727 PMC4806230

[4] Kriesell HJ, Aubin T, Planas‐Bielsa V, Benoiste M, Bonadonna F, Gachot‐Neveu H, et al. Sex identification in King Penguins *Aptenodytes patagonicus* through morphological and acoustic cues. Ibis. 2018;160(4):755–68. doi: 10.1111/ibi.12577

[5] Puts DA, Hill AK, Bailey DH, Walker RS, Rendall D, Wheatley JR, et al. Sexual selection on male vocal fundamental frequency in humans and other anthropoids. Proc Biol Sci. 2016;283(1829):20152830. doi: 10.1098/rspb.2015.2830 27122553 PMC4855375

[6] Ghitza O, Greenberg S. On the possible role of brain rhythms in speech perception: intelligibility of time-compressed speech with periodic and aperiodic insertions of silence. Phonetica. 2009;66(1–2):113–26. doi: 10.1159/000208934 19390234

[7] Nakamura A, Seiyama N, Ikezawa R, Takagi T, Miyasaka E. Real time speech rate converting system for elderly people. In: Proceedings of ICASSP ’94. IEEE International Conference on Acoustics, Speech and Signal Processing. II/225-II/228. doi: 10.1109/icassp.1994.389678

[8] Coupé C, Oh YM, Dediu D, Pellegrino F. Different languages, similar encoding efficiency: comparable information rates across the human communicative niche. Sci Adv. 2019;5(9):eaaw2594. doi: 10.1126/sciadv.aaw2594 32047854 PMC6984970

[9] Banse R, Scherer KR. Acoustic profiles in vocal emotion expression. 1996. 10.1037//0022-3514.70.3.614 8851745

[10] Giraud A-L, Poeppel D. Cortical oscillations and speech processing: emerging computational principles and operations. Nat Neurosci. 2012;15(4):511–7. doi: 10.1038/nn.3063 22426255 PMC4461038

[11] Ghazanfar AA, Hauser MD. The auditory behaviour of primates: a neuroethological perspective. Curr Opin Neurobiol. 2001;11(6):712–20. doi: 10.1016/s0959-4388(01)00274-4 11741023

[12] Drăgănoiu TI, Nagle L, Kreutzer M. Directional female preference for an exaggerated male trait in canary (*Serinus canaria*) song. Proc Biol Sci. 2002;269(1509):2525–31. doi: 10.1098/rspb.2002.2192 12573066 PMC1691196

[13] Blumstein DT, Armitage KB. Alarm calling in yellow-bellied marmots: I. The meaning of situationally variable alarm calls. Anim Behav. 1997;53(1):143–71. doi: 10.1006/anbe.1996.0285

[14] Bergman TJ, Beehner JC, Painter MC, Gustison ML. The speech-like properties of nonhuman primate vocalizations. Anim Behav. 2019;151:229–37. doi: 10.1016/j.anbehav.2019.02.015

[15] Pereira AS, Kavanagh E, Hobaiter C, Slocombe KE, Lameira AR. Chimpanzee lip-smacks confirm primate continuity for speech-rhythm evolution. Biol Lett. 2020;16(5):20200232. doi: 10.1098/rsbl.2020.0232 32453963 PMC7280036

[16] Risueno-Segovia C, Hage SR. Theta synchronization of phonatory and articulatory systems in marmoset monkey vocal production. Curr Biol. 2020;30:4276-4283.e3. 10.1016/j.cub.2020.08.019 32888481

[17] Suthers RA, Fitch WT, Fay RR, Popper AN. Vocal production by terrestrial mammals: Source, filter, and function. Vertebrate sound production and acoustic communication. Cham: Springer International Publishing; 2016. p. 229–59.

[18] Forrest TG. From sender to receiver: Propagation and environmental effects on acoustic signals. Am Zool. 1994;34:644–54.

[19] Ey E, Fischer J. The “acoustic adaptation hypothesis”—a review of the evidence from birds, anurans and mammals. Bioacoustics. 2009;19(1–2):21–48. doi: 10.1080/09524622.2009.9753613

[20] Krams I, Krama T, Freeberg TM, Kullberg C, Lucas JR. Linking social complexity and vocal complexity: a parid perspective. Philos Trans R Soc B. 2012;367:1879–91. 10.1098/rstb.2011.0222 PMC3367703 22641826

[21] Bürkner P-C. brms : an r package for bayesian multilevel models using Stan. J Stat Soft. 2017;80(1). doi: 10.18637/jss.v080.i01

[22] Beaulieu-Laroche L, Brown NJ, Hansen M, Toloza EHS, Sharma J, Williams ZM, et al. Allometric rules for mammalian cortical layer 5 neuron biophysics. Nature. 2021;600(7888):274–8. doi: 10.1038/s41586-021-04072-3 34759318 PMC8665137

[23] Buzsáki G, Logothetis N, Singer W. Scaling brain size, keeping timing: evolutionary preservation of brain rhythms. Neuron. 2013;80:751–64. 24183025 10.1016/j.neuron.2013.10.002 PMC4009705

[24] Knyazev GG. EEG delta oscillations as a correlate of basic homeostatic and motivational processes. Neurosci Biobehav Rev. 2012;36(1):677–95. doi: 10.1016/j.neubiorev.2011.10.002 22020231

[25] Raccuglia D, Huang S, Ender A, Heim M-M, Laber D, Suárez-Grimalt R, et al. Network-specific synchronization of electrical slow-wave oscillations regulates sleep drive in *Drosophila*. Curr Biol. 2019;29(21):3611-3621.e3. doi: 10.1016/j.cub.2019.08.070 31630955

[26] Morillon B, Arnal LH, Schroeder CE, Keitel A. Prominence of delta oscillatory rhythms in the motor cortex and their relevance for auditory and speech perception. Neurosci Biobehav Rev. 2019;107:136–42. doi: 10.1016/j.neubiorev.2019.09.012 31518638

[27] Gröchenig K. Time-frequency analysis and the uncertainty principle. Foundations of time-frequency analysis. Boston, MA: Birkhäuser Boston; 2001. p. 21–36.

[28] Dogonasheva O, Doelling K, Zakharov D, Giraud AL, Gutkin B. A brain-rhythm based computational framework for semantic context and acoustic signal integration in speech processing. Neuroscience. 2024. doi: 10.1101/2024.01.17.575994

[29] Andics A, Gábor A, Gácsi M, Faragó T, Szabó D, Miklósi Á. Neural mechanisms for lexical processing in dogs. 4. 10.1126/science.aaf3777 27576923

[30] Déaux EC, Piette T, Gaunet F, Legou T, Arnal L, Giraud A-L. Dog-human vocal interactions match dogs’ sensory-motor tuning. PLoS Biol. 2024;22(10):e3002789. doi: 10.1371/journal.pbio.3002789 39352912 PMC11444399

[31] Boesch C, Crockford C. Call combinations in wild chimpanzees. Behav. 2005;142:397–421.

[32] Collier K, Townsend SW, Manser MB. Call concatenation in wild meerkats. Anim Behav. 2017;134:257–69. doi: 10.1016/j.anbehav.2016.12.014

[33] Coye C, Ouattara K, Zuberbühler K, Lemasson A. Suffixation influences receivers’ behaviour in non-human primates. Proc Biol Sci. 2015;282(1807):20150265. doi: 10.1098/rspb.2015.0265 25925101 PMC4424650

[34] Engesser S, Ridley AR, Townsend SW. Meaningful call combinations and compositional processing in the southern pied babbler. Proc Natl Acad Sci U S A. 2016;113(21):5976–81. doi: 10.1073/pnas.1600970113 27155011 PMC4889383

[35] Wilson DE, Mittermeier RA, Cavallini P. Handbook of the mammals of the world. Barcelona: Lynx Edicions; 2009.

[36] del Hoyo J, Elliott A, Sargatal J, Cabot J. Handbook of the birds of the world. Barcelona: Lynx Edicions; 1992.

[37] Rombaut LMK, Capp EJR, Cooney CR, Hughes EC, Varley ZK, Thomas GH. Allometric conservatism in the evolution of bird beaks. Evol Lett. 2021;6(1):83–91. doi: 10.1002/evl3.267 35127139 PMC8802239

[38] Guilford T, Dawkins MS. Receiver psychology and the evolution of animal signals. Anim Behav. 1991;42(1):1–14. doi: 10.1016/s0003-3472(05)80600-1

[39] Tilsen S, Johnson K. Low-frequency Fourier analysis of speech rhythm. J Acoust Soc Am. 2008;124(2):EL34-9. doi: 10.1121/1.2947626 18681499 PMC5570052

[40] Bowling DL, Garcia M, Dunn JC, Ruprecht R, Stewart A, Frommolt K-H, et al. Body size and vocalization in primates and carnivores. Sci Rep. 2017;7:41070. doi: 10.1038/srep41070 28117380 PMC5259760

[41] Benson RBJ, Mannion PD. Multi-variate models are essential for understanding vertebrate diversification in deep time. Biol Lett. 2012;8(1):127–30. doi: 10.1098/rsbl.2011.0460 21697163 PMC3259948

[42] Katoh K, Standley DM. MAFFT multiple sequence alignment software version 7: improvements in performance and usability. Mol Biol Evol. 2013;30(4):772–80. doi: 10.1093/molbev/mst010 23329690 PMC3603318

[43] Sueur J, Aubin T, Simonis C. Seewave, a free modular tool for sound analysis and synthesis. Bioacoustics. 2008;18(2):213–26. doi: 10.1080/09524622.2008.9753600

[44] Vehtari A, Gelman A, Gabry J. Practical Bayesian model evaluation using leave-one-out cross-validation and WAIC. Stat Comput. 2017;27:1413–32.

[45] Grabowski M, Kopperud BT, Tsuboi M, Hansen TF. Both diet and sociality affect primate brain-size evolution. Syst Biol. 2023;72:404–18. 36454664 10.1093/sysbio/syac075 PMC10275546

[46] Yu G, Smith DK, Zhu H, Guan Y, Lam TT. ggtree : an r package for visualization and annotation of phylogenetic trees with their covariates and other associated data. Methods Ecol Evol. 2016;8(1):28–36. doi: 10.1111/2041-210x.12628

[47] Anikin A. Soundgen: an open-source tool for synthesizing nonverbal vocalizations. Behav Res Methods. 2019;51(2):778–92. doi: 10.3758/s13428-018-1095-7 30054898 PMC6478631

[48] Højsgaard S, Halekoh U. doBy: groupwise statistics, lsmeans, linear estimates, utilities. 2006.

[49] Bates D, Mächler M, Bolker B, Walker S. Fitting linear mixed-effects models using lme4. J Stat Soft. 2015;67.

[50] Bartoń K. MuMIn: multi-model inference. 2010.

[51] Wickham H. Ggplot2: elegant graphics for data analysis. 2nd ed. Switzerland: Springer; 2016.

