# Correction: Animal acoustic communication has a conserved optimal rhythm within the neural delta range

**DOI:** 10.1371/journal.pbio.3003980

**Published:** 2026-08-31

**Authors:** Theophane Piette, Chundra Cathcart, Chiara Barbieri, Keesha Martin Ming, Didier Grandjean, Balthasar Bickel, Eloïse Déaux, Anne-Lise Giraud

The third author’s name is spelled incorrectly. The correct name is: Chiara Barbieri.

The fourth author’s initials appear incorrectly in the citation. The correct citation is: Piette T, Cathcart C, Barbieri C, Ming KM, Grandjean D, Bickel B, et al. (2026) Animal acoustic communication has a conserved optimal rhythm within the neural delta range. PLoS Biol 24(6): e3003798. https://doi.org/10.1371/journal.pbio.3003798.

## References

[1] Piette T, Cathcart C, Barbieri C, Martin Ming K, Grandjean D, Bickel B, et al. Animal acoustic communication has a conserved optimal rhythm within the neural delta range. PLoS Biol. 2026;24(6):e3003798. doi: 10.1371/journal.pbio.3003798 42263115 PMC13249164

