# Supplementary figures and images for: Animal acoustic communication has a conserved optimal rhythm within the neural delta range
Source: PLoS Biol. 2026 Jun 9;24(6):e3003798. doi: 10.1371/journal.pbio.3003798 (PMC13249164; doi:10.1371/journal.pbio.3003798)

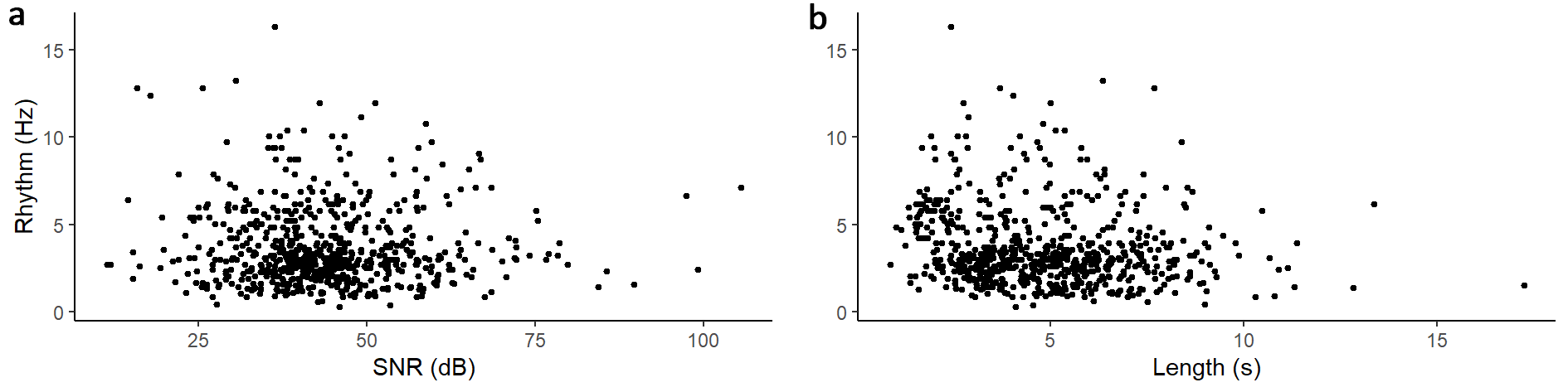

Supplement: S1 Fig — a) Rhythm (Hz) as a function of signal-to-noise ratio (SNR) showing the absence of relationship between SNR and Rhythm (t = −0.21, p = 0.84, R2 = 0.02). b) Rhythm (Hz) as a function of recording length showing the absence of relationship between sequence length and rhythm (t = −1.45, p = 0.15, R2 = 0.02). The data underlying this Figure can be found in https://zenodo.org/records/19816728. (TIF) [file pbio.3003798.s002.tif]

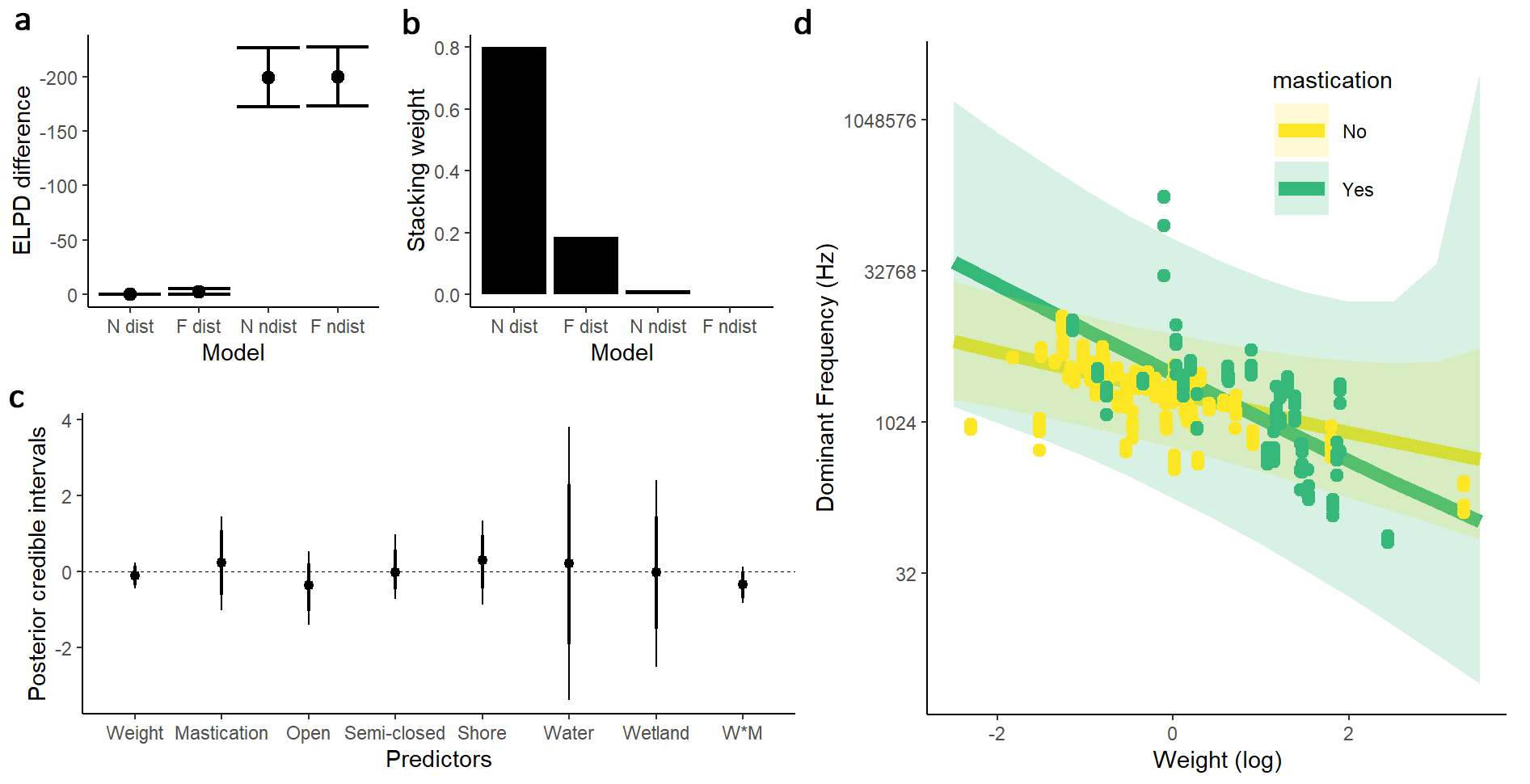

Supplement: S2 Fig — a) Leave-one-out expected log pointwise density difference (ELPD) between the full and the null distributional models. b) Stacking weight of the models. c) Posterior credible interval (95% and 85%) of the full model. d) Dominant frequency plotted on a logarithmic scale as a function of log-transformed weight with predicted slopes from the full distributional model and their standard error. The data underlying this Figure can be found in https://zenodo.org/records/19816728. (TIF) [file pbio.3003798.s003.tif]

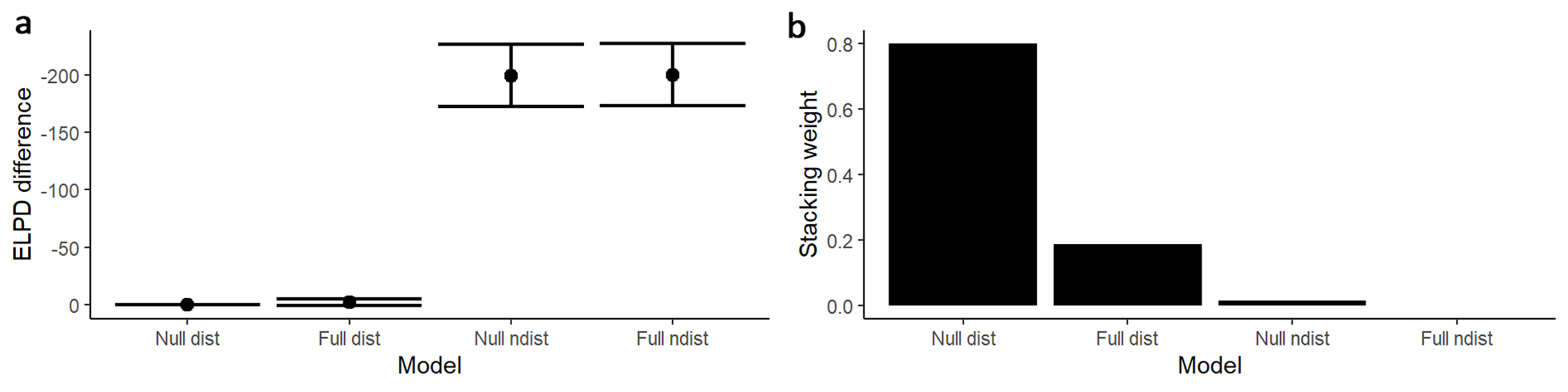

Supplement: S3 Fig — a) Leave-one-out expected log pointwise density difference (ELPD) between the null distributional (“dist”) model and the others (“ndist” for “non-distributional, modeling only the mean”). b) Stacking weight of the models. The data underlying this Figure can be found in https://zenodo.org/records/19816728. (TIF) [file pbio.3003798.s004.tif]

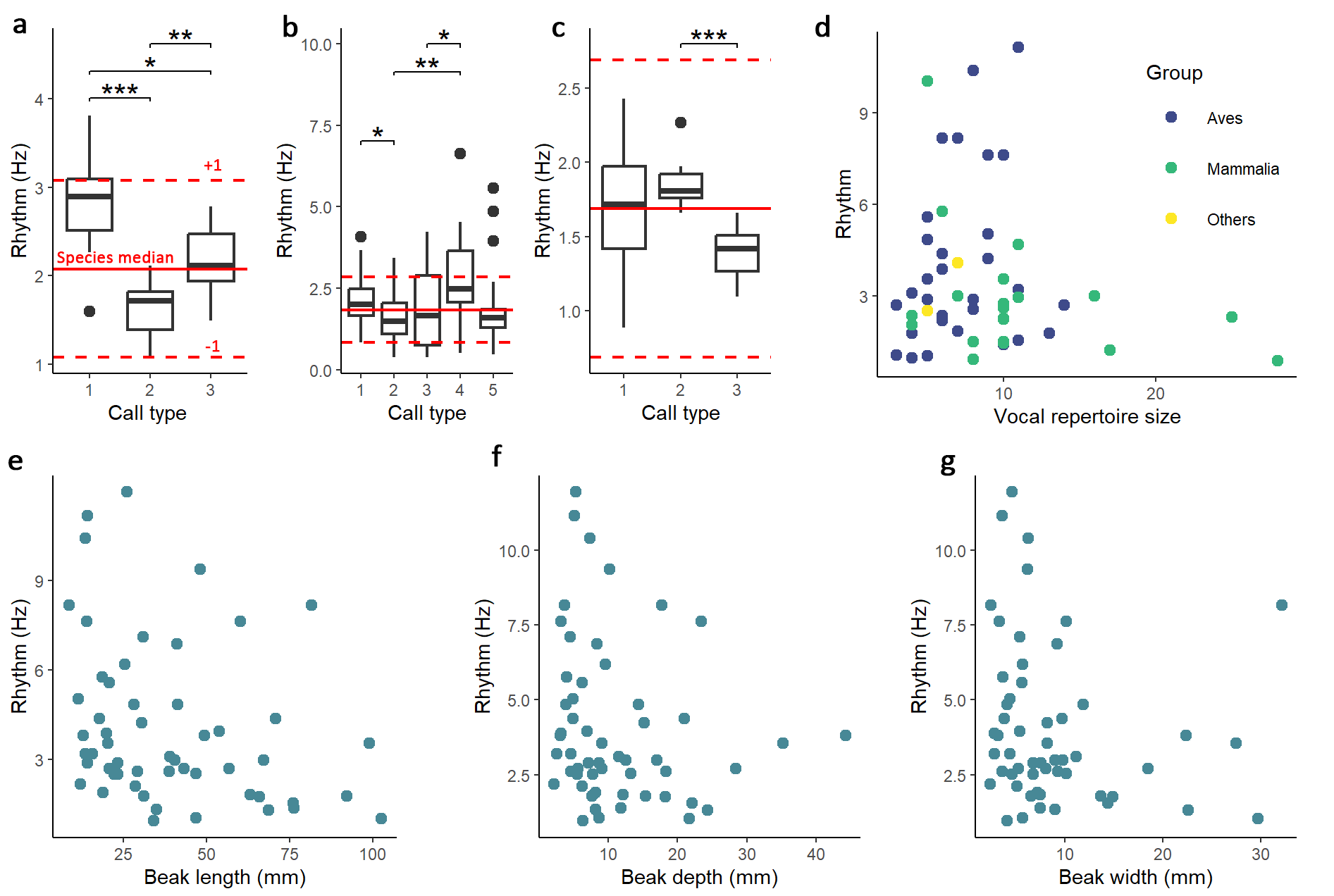

Supplement: S5 Fig — a) Rhythm in sequences of different call types (1 = affiliative grunt, 2 = scream, 3 = threat grunt) in olive baboons (Papio anubis). b) Rhythm in sequences of different call types (1 = bark, 2 = growl, 3 = howl, 4 = snarl, 5 = whine) in dogs (Canis lupus familiaris). c) Rhythm in sequences of different call types (1 = alarm call, 2 = flight call, 3 = song) in Eurasian stone-curlews (Burhinus oedicnemus). d) Rhythm plotted as a function of vocal repertoire size. e) Rhythm plotted as a function of beak length in birds. f) Rhythm plotted as a function of beak depth in birds. g) Rhythm plotted as a function of beak width in birds. The data underlying this Figure can be found in https://zenodo.org/records/19816728. (TIF) [file pbio.3003798.s006.tif]
