# Supplementary material for: Animal acoustic communication has a conserved optimal rhythm within the neural delta range
Source: PLoS Biol. 2026 Jun 9;24(6):e3003798. doi: 10.1371/journal.pbio.3003798 (PMC13249164; doi:10.1371/journal.pbio.3003798)
Supplement: Attachment — Submitted filename: Comments for PBIOLOGY-D-25-02159_R2.pdf [file pbio.3003798.s010.pdf]

The manuscript has been significantly improved. Information added about how recordings were selected cleared my question before. The authors have also focused on birds and mammals for the phylogenetic history analysis and so most of my earlier questions and criticisms about phylogenetic representation and different mechanisms of rhythmic generation and detection in invertebrates and vertebrates no longer apply. The main conclusion remains the same and stands stronger now.

However, the issues that those hypotheses species specific selective pressures are vertebrate biased continue to exist. I agree that it is important to include animals other than birds and mammals in the survey of the acoustic data to show that a low rhythm is widespread among acoustically communicating animals. I also think focusing on birds and mammals only for the phylogenetic pattern is a wise choice. Doing so does not undermine how novel and exciting the new hypothesis is and make the possibility sounder and more rigorous. Along the same line, I suggest the authors to run the Bayesian model using only birds and mammals and move model output with everything included to the supplementary. Many mechanisms the authors mention in the Introduction, such as relationship with articulators inherited from mastication, breathing rate, and social complexity apply to vertebrates and vocally communicating animals. Such mechanisms do not apply to animals that produce sound by, for example, rubbing wings or vibrating substrates with legs, for which sounds production does not rely on air exchange. In addition, data about social complexity is highly biased towards eusocial vertebrates and for many other taxa, it is difficult to distinguish whether there is lack of social complexity or lack of data. New data from what we used to think as simple, non-social animals keep surprising us. Although the model itself is robust to low sample size in some groups, the rationale to include them in the first place is not as sound. By using a two-tiered presentation of the results, those with sound mechanistic rationale and rich and high-quality data in the main results and the rest in the supplementary to show the potential of the model, the author can support their point on a much more solid ground. Otherwise, anyone who studies acoustic communication in insects may have problem with the rationales in the Introduction. Because the idea about delta oscillation and signal reception is novel enough, whether they apply to insects or not does not make it or break it. Even if the mechanism turns out to be not true in, for example, fish and insects, I don’t think this makes the idea any less exciting.
