# Supplementary material for: Animal acoustic communication has a conserved optimal rhythm within the neural delta range
Source: PLoS Biol. 2026 Jun 9;24(6):e3003798. doi: 10.1371/journal.pbio.3003798 (PMC13249164; doi:10.1371/journal.pbio.3003798)
Supplement: Attachment — Submitted filename: Response_Reviewver_Plos_Biology.docx [file pbio.3003798.s009.docx]

**REVIEWERS' COMMENTS:**


**Reviewer #1:**

This is a very good Discovery Report, clearly presented, with the right amount of data backing up the main claim, and the right level of Discussion. I am happy to recommend publication.

Concerning the Discussion, I was wondering if the authors would find the following findings noteworth in the context of their Discussion (<https://www.biorxiv.org/content/10.1101/2024.12.02.626521v1>)

We thank the reviewer for their very positive assessment of our work and their recommendation for publication.

**Reviewer #2:**

This manuscript presents an observation of an overrepresentation of temporal tempo below 5 Hz in acoustic signals across animal phyla and subsequently tests four non-mutually exclusive hypotheses for the evolution of the observed pattern. These hypotheses include both ultimate explanations (signal efficacy, i.e., transmission in the environment and social function) and proximate explanations (neural mechanism and physiological constraints).

The questions asked in this manuscript are both intriguing and novel and the scope of interest is wide. Temporal structures are a ubiquitous feature of acoustic signals of animals. The question whether temporal rhythm is distributed randomly or does its distribution show some pattern across animals is an intriguing and novel one. If a pattern is observed, asking what gives rise to the pattern should be of interest for a wide range of fields, including neuroscience, animal behavior, and evolutionary biology. Among the four hypotheses tested, the latter three (physiological constraints, environmental influence, and social influence) are not new. These hypotheses have been proposal and tested in similar contexts. The first hypothesis about a deeply conserved neural mechanism is novel. I applaud the authors' effort to try to bring together neurobiology, behavior, and evolution together. Such a perspective may foster cross talk among fields that do not talk to each other enough
and has the potential to generate transformative insights.

The idea about rhythmicity in acoustic signals and delta oscillation in the brain is bold. This inherently means the idea comes with some degree of forward-projecting, with more gaps in the current evidence chain than some other less bold ideas. I believe these alone should not be the reason to kill bold ideas. The key lies in whether our current theory and empirical data points to a promising path for further investigation. With all these in mind, this manuscript falls short of demonstrating careful and thorough examination of existing theory and data and construction of a solid foundation for the novel idea.

We sincerely thank the reviewer for their thoughtful and encouraging comments. We are grateful for their recognition of the novelty and interdisciplinary scope of this study, and we appreciate the opportunity to clarify the theoretical reasoning underlying our hypothesis. In the revised Discussion (lines 180-225), we have expanded the conceptual background to better explain the exploratory nature of our work and the rationale connecting our findings to known neurobiological mechanisms.

This study was conceived as an exploratory investigation rather than a test of predetermined hypotheses. Our goal was to examine whether the slow temporal modulations, typically in the theta range, that play a key role in human speech processing would also be found in animal acoustic communication. An important aspect of our study is the fundamental difference between the pitch of communication signals and their envelope, the former scaling with the animal weight (size), the latter being independent and following a conservation law.

Our analyses identified a conserved rhythm centered around 2.9 Hz across a wide range of taxa. Although we only explored the low-end of the modulation spectrum (0.1 to 20Hz), a common rhythm in the delta range emerged empirically from the data and guided our interpretation. That communication rhythms did not scale with the animal weight is one the main (and surprising) findings of the study. Phylogenetic analyses showed that rhythm evolution across species follows an Ornstein–Uhlenbeck (OU) process, indicating strong stabilizing selection toward a common optimum rather than random divergence. If acoustic rhythm were mainly driven by species-specific or ecological factors such as environment, biomechanics, or social context, we would expect high variability and no consistent convergence across lineages. Instead, the OU pattern points to a deeply conserved constraint acting across the tree of life. The statistical significance of this finding shows that the number of studied species is sufficient to at least formulate a novel hypothesis about a conserved process.

Given this conservation, the question becomes what could drive such stability. Morphological and environmental factors vary widely, but the physiological properties of neurons, especially their conductance, are highly conserved. The rhythm we observed (~2.9 Hz) interestingly lies within the delta frequency range (1–4 Hz), a band universally present across animal taxa and known to structure slow information integration in the nervous system. In mammals, delta oscillations in auditory cortices (for instance, in bats, dogs, and humans) have been shown to track and segment acoustic input and to interact with higher-frequency gamma oscillations through phase–amplitude coupling (PAC).

From this perspective, we hypothesize a two-timescales processing of acoustic information by the brain (complex neural systems). In this model, delta oscillations provides a long integration window that supports signal identification, segmentation and cross-modal integration, allowing the receiver to group acoustic events into coherent and meaningful percepts. In addition, faster oscillation (most likely low-gamma one), characterized by much shorter time windows, could support rapid detection and discrimination of transient features within those segments. The current results do not provide information about this timescale, but a vast literature shows it is also conserved and coupled to slower rhythms (Lakatos ? and more). For instance, we find such delta/low-gamma articulation in baboons (in prep). The interaction between these two rhythms could ensure that fast sensory analysis remains temporally aligned with slower integrative processes, allowing communication to be both precise and intelligible. The conserved tempo around 2.9 Hz may therefore correspond to an intrinsic rate at which neuronal populations synchronize to efficiently integrate sensory (auditory) information, and the timescale at which communication is best received and produced.

That our analyses and interpretations focus on the slow temporal dynamics that are conserved across species does not imply that we discard the existence or importance of faster timescales, but rather that this study represents a first step in exploring the slow, integrative end of the neural processing spectrum. Future neurophysiological investigations will examine how these faster oscillations interact with the delta rhythm to shape perception and communication. In on-going work, we explore roughness in bird vocalisations..

The conclusion that there is an overrepresentation of optimum rhythmic tempo in acoustic signals below 5 Hz across animals lack validity. First, this conclusion come from too low a sample size. Specifically, besides birds and mammals, the other classes are represented by 4 or even only 1 species. Even the number of bird and mammal species are not very high. Such a sample size is extremely biased in terms of taxonomic group and it is difficult to imagine this small sample can truthfully reflect the diversity in taxonomic groups and temporal structures in acoustic signals. Second, there is no detailed description about how these species and acoustic samples are chosen. It is thus impossible to determine whether the samples are chosen with a reasonable criteria. The fact that exceptions are plentiful, but the selected data show a very strong pattern raises the possibility of cherry-picking.

We thank the reviewer for pointing out that our selection process and sampling criteria were not described with sufficient clarity in the initial version of the manuscript. Data collection in fact followed a strict protocol, now fully detailed in the Methods section (see lines 272-286). Our plan was to obtain acoustic recordings from one representative species per infraorder of tetrapods, based on the most up-to-date phylogenetic classifications. For each order, we screened public databases and ranked species by the number of available recordings. The first species fulfilling the following criteria was then selected: at least five clean recordings (non-noisy, with a single individual vocalizing at a time) from different individuals. Once such a species was identified, we proceeded to the next infraorder. When no species from a given infraorder was available in public databases, we extended the search to private databases and directly contacted researchers. If no data could be obtained, that infraorder was excluded. Our selection process was thus constrained by the availability of clean, high-quality recordings that met these strict criteria. Our objective was to perform a broad comparative analysis across tetrapods, and our sampling strategy was therefore designed to reflect the natural distribution of taxonomic diversity rather than to artificially equalize species numbers across groups. As a result, our dataset includes 58 bird species and 28 mammal species (approximately 68% birds and 32% mammals), proportions that closely match the natural distribution of species richness between these two clades (around 18,000 bird species; Barrowclough et al., 2016, and 6,759 mammal species; Burgin et al., 2025, representing 73% birds and 27% mammals). Balancing the dataset numerically would have artificially overrepresented mammals relative to their actual diversity.

For example, in insects, the repetitive elements in the song could occur at high rates,
some are a magnitude higher (Grobe et al., 2012; Skovmand and Boel Pedersen, 1983; Spooner, 1964; Ulagaraj, 1976; Ulagaraj and Walker, 1975; von Schilcher, 1976; Walker, 2000). What are the reasons the one insect selected falls within the so-called optimum tempo range but these species whose tempo falls outside the range were not included? Third, acoustic sample were collected from multiple sources, including some online video platforms where it is difficult to standardize the recording condition and quality. For example, if the acoustic samples were recorded in an environment where there were noise sources that show rhythmic pattern, how can the focal signal and these noises be teased apart?

In this study, we focused on amplitude variations and computed the acoustic envelope up to 20 Hz . This choice was motivated by our aim to characterize the slow temporal dynamics in acoustic communication. We do not claim that the rhythm observed at 2.9 Hz is the only relevant timescale in animal vocalizations. As the reviewer correctly notes, insect signals often contain faster, nested rhythms, such as pulses grouped into chirps or verses. We explicitly acknowledge this in the discussion (lines 210-225), where we point out the likely necessity of faster processing rhythms, and we even suggest that a promising line of inquiry is to investigate how slow and fast timescales interact to optimize acoustic communication.

That the retained species falls within the so-called “optimum tempo” range is therefore the outcome of this systematic procedure, rather than the result of any intentional exclusion of species with faster temporal structures. Finally, with respect to the heterogeneity of recording sources, we took particular care to ensure data quality and comparability. Only recordings with minimal background noise and a single focal individual were retained. Any samples containing rhythmic background noise or ambiguous signals were excluded. This rigorous filtering minimized the risk of confounding effects and ensured that the extracted acoustic envelopes reliably reflected the focal signal across different sources.

In ectotherm animals, it is well-known that the temporal rhythm of the acoustic signal change with ambient temperature. For endotherm animals, the body temperature may vary across species and the temporal pattern of the acoustic signals may also vary with body temperature. How is temperature standardized across samples? Because the models cannot outperform input data, the conclusions from models with problematic input data are thus unsound. And as this conclusion forms the foundation of the hypotheses tested, the entire study is built on a shaky ground.

Going forward, there can be a few options. My suggestion is to focus on birds and mammals only for a few reasons. First, the most novel hypothesis about delta oscillation are best fit for birds and mammals (see detailed reasons below in my comments about this hypothesis). Second, with the magnitude of taxonomic diversity of invertebrates and especially insects, it is an extremely difficult task to collect sound samples and data for environment, social complexity and both parameters that can be representative of this group. If the authors focus on just birds and mammals, it is much more manageable. Next, I suggest the authors to:

(1)     Come up with universal and standardized protocol and criteria for data search and filter. There needs to be a standardized protocol for systematically searching the literature and databases. This protocol should specifically address both coverage of the search and measures to mitigate bias in data inclusion. There also should be universal criteria for selecting and processing sound samples.

(2)     Increase sample size such that the sample size is sufficient to represent the taxonomic group the authors intend to focus on

We thank the reviewer for this comment and acknowledge that outside birds and mammals the coverage of other tetrapod species is limited. Following the reviewer’s suggestion, we have now focused our main analyses on birds and mammals, which yielded similar results: a conserved rhythm of acoustic signals in the delta range (mean rhythm = 2.73 Hz; 95% HDI [0.45–4.99]). This pattern also holds within each clade analyzed separately (Mammals: 2.3 Hz, 95% HDI [0.34–4.8]; Birds: 2.8 Hz, 95% HDI [0.34–5.6]), confirming that the observed relationship is not driven by an overrepresented taxon, but rather conserved across both groups.

The full model including all other taxa has been moved to the Supplementary Material, where these additional species are presented as complementary evidence. We believe this is an important piece of information to guide future explorations. Although less represented, these groups provide valuable support suggesting that the rhythmic constraints identified in our main analysis may extend more broadly across tetrapods, potentially reflecting an older evolutionary origin.

Second, I think the hypothesis about delta oscillation playing a role in the generation of a certain rhythmic tempo is very intriguing and bold but I have several concerns about this hypothesis. First, the range of taxonomic group the authors proposed may be too wide. The neural mechanism for the generation of rhythmic pattern in songs of insects, frogs and fish have been well studied. In all of these animals, the central pattern generator is a major driver of the rhythmic pattern (Bass, 2014; Clyne and Miesenböck, 2008; Grobe et al., 2012; Rhodes et al., 2007; Schöneich and Hedwig, 2011, 2017; Skovmand and Boel Pedersen, 1983; Spooner, 1964; Ulagaraj, 1976; Ulagaraj and Walker, 1975; von Schilcher, 1976; Walker, 2000). I may be wrong but so far, I have not found any evidence that delta oscillation plays any role in the output of central pattern generators besides potential entrain or modulation in these animals. This raises the question about whether the proposed mechanism
apply to these taxonomic groups or not. If there are good evidence showing that the delta rhythm determines the output of singing CPG in these animals, the authors should add the evidence to the manuscript.

We thank the reviewer for this comment, which highlights a potential misunderstanding that could indeed arise from our original wording. In no way do we suggest that delta oscillations are directly involved in the neural generation of acoustic signals, nor that they are involved in the well-established role of central pattern generators (CPGs). The hypothesis we advance is that delta oscillations are critical for acoustic signal processing in the receiver’s brain. If acoustic signals are structured (produced) at rates that match auditory delta oscillations, the receiver’s brain would be better tuned to extract and process information at these timescales. This could in turn explain why such temporal rates have been conserved across diverse taxa, despite the diversity of motor mechanisms underlying signal production, hence the interest of exploring species that do not vocalize such as insects, fishes or amphibians. In light of the reviewer’s comment, we have carefully revised the Discussion (see lines 199-217) to more clearly emphasize the role of delta oscillations in signal processing rather than in signal production, thereby avoiding ambiguity.

Second, just because the tempo of two phenomena coincide does not necessarily imply causality. To establish causality, I expect a more solid logic chain supported by current theory and empirical data. The links on this chain may not be all connected yet but all (or at least most) steps of this logic chain needs to be clearly presented to make this hypothesis more plausible. Because behavioral and neural organizations represent different levels of organizations that can be independent of each other (Katz, 2016), it is entirely possible these two things have nothing to do with each other or the relationship is not what appears on the surface. And a minor point, when the hypothesis was first presented, the authors mentioned theta, instead of delta, rhythm. This however, does not mean there is no wisdom to the idea. I suggest the authors to present a more concrete mechanism that can be backed up by theory and data.

We thank the reviewer for raising this point. We do not imply causality from the simple match between acoustic rhythm and neural oscillatory ranges. In the revised discussion, we clarify why a conserved slow rhythm naturally leads us to consider slow neural integration mechanisms.The rhythm we identify across species (mean 2.8 Hz) falls within the delta band, a slow oscillation that is itself highly conserved across mammals, birds, reptiles and insects. Delta rhythms support slow perceptual integration and active sensing, making them a plausible neural timescale for grouping acoustic information. In our framework, delta would support the integration of acoustic structure, while a faster range (likely low-gamma) would support rapid detection and discrimination. This organisation aligns with the Fourier uncertainty principle and with psychophysical evidence that restoring slow modulations is critical for speech intelligibility. Importantly, dogs also process human speech at slow, delta rates, rather than at the syllabic rhythm, demonstrating that delta-based integration operates across species. Together, these converging observations support the plausibility of our hypothesis.

Another major problem is that support for this hypothesis is through process of elimination and there is no actual test of this hypothesis. This significantly weakens the validity of the conclusion because the four hypotheses are not exhaustive and the tests of the other hypothesis are problematic such that they cannot be confidently rejected (see comments below).

The other hypotheses and their tests have various problem as well. First, the hypothesis about morphological and physiological constraints is very vertebrate-centric. It was not explicitly stated but I'm guessing that weight and mastication status (and it seems beak morphology for birds too) are the two factors used to test this hypothesis. Invertebrates produce sounds by entirely different mechanisms such that these factors do not apply to them. Even for vertebrates, the constraints can be trade-offs in vocal performance that these factors cannot capture. For example, in birds, it is well known that there is a constraint in vocal performance such that there are tradeoffs between trill rate and frequency band width and learning accuracy (Ballentine et al., 2004; Podos et al., 2004).

We thank the reviewer for these detailed comments and for highlighting the distinction between species-specific factors and broader evolutionary mechanisms. We fully acknowledge that variables such as body weight, living environment, or social dynamics can modulate acoustic rhythm within a given species. To directly address this point, we performed a supplementary analysis on the subset of species for which contextual data were available. This analysis confirmed that behavioral context does influence rhythm, but only within a narrow range of variation (approximately ±1 Hz around the species’ median rhythm). This suggests that, while context can fine-tune rhythm locally, it does not substantially alter the characteristic tempo of a species.

Our Bayesian multilevel model, by design, cannot fully exclude such local influences acting at the microevolutionary scale. However, our results, derived from a comparative phylogenetic framework, address a fundamentally different question: the existence of a macroevolutionary constraint shaping rhythmic structure across distant taxa. The key finding is that the evolution of acoustic rhythm across species is best described by an Ornstein–Uhlenbeck (OU) process rather than by a Brownian motion process. This distinction is crucial, as a Brownian process would indicate that rhythm evolves through unconstrained, gradual divergence driven by species-specific factors such as morphology or environment. Instead, the OU pattern, with 93% of phylogenetic half-lives below one, reveals strong stabilizing selection and rapid evolutionary reversals toward a conserved optimum rhythm. This pattern indicates that, even though local factors may introduce small variations, they do not explain the overall evolutionary trajectory of rhythm across lineages.

In summary, if rhythm were predominantly shaped by external or transient ecological pressures, we would expect random, noise-like divergence and no clear evolutionary pull toward a common rhythm. The fact that we observe the opposite, a consistent conservation toward a shared rhythmic range across taxa, suggests the presence of an endogenous, evolutionarily stable constraint that transcends taxonomic boundaries. We interpret this as evidence for a conserved perceptual or neural mechanism underlying rhythm processing in animals. We have clarified this interpretation in the revised discussion (lines 180-198), explicitly stating that while our approach cannot discard the influence of local, short-term factors at the microevolutionary level, the phylogenetic pattern itself effectively rejects their dominance at the macroevolutionary scale. This study therefore represents an important first step in linking bioacoustics and neuroscience to understand the fundamental mechanisms governing rhythm in animal communication.

The hypothesis about environmental constraints, or signal efficacy is valid but the citation the authors cited (citation 18) is not a good one. Please cite a better source. There are many papers from behavioral ecology on this topic. However, data used for testing the hypothesis is crude and the accuracy questionable as these data mainly come from two books that were not peer reviewed. It is unclear how the authors of these books collected these data and if multiple sources exist, how were data selected. This criticism applies to tests of all three hypotheses except for the first one.

We thank the reviewer for this comment. and have updated citation 18 with (T. G. Forrest, From Sender to Receiver: Propagation and Environmental Effects on Acoustic Signals. Am Zool 34, 644–654 (1994)). Regarding the reviewer’s concerns about data quality, we would like to clarify that the Handbook of the Birds of the World (HBW) and the Handbook of the Mammals of the World (HMW) are internationally recognized reference works widely used by the scientific and conservation communities. Both series are produced in collaboration with hundreds of taxonomic, ecological, and behavioral experts and systematically cite the primary literature from which their data are drawn. We acknowledge that data on body weight and habitat inevitably vary when collated across thousands of species, but HBW and HMW represent the most comprehensive, consistently curated, and globally accepted datasets available for large-scale comparative analyses. For this reason, they have been used as authoritative references in numerous peer-reviewed studies in ecology, behavior, and conservation.

Last, I fail to understand the rationale for the hypothesis about rhythm and social complexity. Social complexity can affect vocal repertoire but how does this inform us about the relationship between social complexity and temporal rhythm? On the other hand, social interactions may very well influence signal design, including temporal rhythm. For example, repetition rate may increase in aggressive contexts. In the context of mate choice, females may prefer either a specific rate or fast rate. Males competing to gain access to mate are selected to perform according to what females prefer. Such influences may be taxonomic- and context-dependent and I'm not sure how a universal factor can capture these relationships.

We thank the reviewer for this comment and have clarified our rationale for the social complexity hypothesis (lines 80-82). Because rhythm limits the rate of information transfer, we reasoned that species with more complex social systems might rely on faster acoustic tempos to communicate more information within a given time frame.

Due to these reasons, the conclusion that these three hypotheses should be rejected is not sound. I suggest the authors to carefully reconsider the hypotheses to make sure they are reasonable and are well backed up by theory and empirical data. Testing these hypotheses, however, may be a much bigger task than what was done in this study. It is doable but a good test needs to be built on good quality data, meticulous scrutiny and curation of the collected data, as well as finding ways to come up with parameters that can both capture the relationship and are universal among all animals tested.

Ballentine, B., J. Hyman, and S. Nowicki. 2004. Vocal performance influences female response to male bird song: an experimental test. Behavioral Ecology, 15:163-168.
Bass, A. H. 2014. Central pattern generator for vocalization: is there a vertebrate morphotype? Current opinion in neurobiology, 28:94-100.
Clyne, J. D., and G. Miesenböck. 2008. Sex-specific control and tuning of the pattern generator for courtship song in Drosophila. Cell, 133:354-363.
Grobe, B., M. M. Rothbart, A. Hanschke, and R. M. Hennig. 2012. Auditory processing at two time scales by the cricket Gryllus bimaculatus. Journal of Experimental Biology, 215:1681-1690.
Katz, P. S. 2016. Evolution of central pattern generators and rhythmic behaviours. Phil. Trans. R. Soc. B, 371:20150057.
Podos, J., S. K. Huber, and B. Taft. 2004. Bird song: the interface of evolution and mechanism. Annu. Rev. Ecol. Evol. Syst., 35:55-87.
Rhodes, H. J., J. Y. Heather, and A. Yamaguchi. 2007. Xenopus vocalizations are controlled by a sexually differentiated hindbrain central pattern generator. Journal of Neuroscience, 27:1485-1497.
Schöneich, S., and B. Hedwig. 2011. Neural basis of singing in crickets: central pattern generation in abdominal ganglia. Naturwissenschaften, 98:1069-1073.
—. 2017. Neurons and Networks Underlying Singing Behaviour, The Cricket as a Model Organism, pp. 141-153: Springer.
Skovmand, O., and S. Boel Pedersen. 1983. Song recognition and song pattern in a shorthorned grasshopper. Journal of comparative physiology, 153:393-401.
Spooner, J. D. 1964. The Texas bush katydid—its sounds and their significance. Animal Behaviour, 12:235-IN4.
Ulagaraj, S. 1976. Sound production in mole crickets (Orthoptera: Gryllotalpidae: Scapteriscus). Annals of the Entomological Society of America, 69:299-306.
Ulagaraj, S., and T. J. Walker. 1975. Response of flying mole crickets to three parameters of synthetic songs broadcast outdoors. Nature, 253:530-532.
von Schilcher, F. 1976. The function of pulse song and sine song in the courtship of Drosophila melanogaster. Animal Behaviour, 24:622-625.
Walker, T. J. 2000. Pulse rates in the songs of trilling field crickets (Orthoptera: Gryllidae: Gryllus). Annals of the Entomological Society of America, 93:565-572.

**Reviewer #3:**

Review of 'A conserved rhythm in animal acoustic communication' (PBIOLOGY-D-25-02159R1)

This manuscript addresses a current issue in animal behavior, cognition and neuroscience, and could potentially be of interest to many readers.  For the most part, the authors have carefully followed protocols for the comparative and phylogenetic method, have explained their quantitative analyses and assumptions, and claim that animal rhythms - in acoustic communication (including human speech and music) - generally fall within a narrow tempo (repetition rate) range and may do so because of fundamental properties of central nervous systems.  Below, I offer recommendations that could sharpen the analyses, and I also note some caveats.

We thank the reviewer for their positive and constructive comments. We appreciate the acknowledgment of the study’s relevance and methodological rigor, and we have carefully considered their recommendations to further refine our analyses and clarify our assumptions.

The authors present a body of results that may support the hypothesis that the typical values of animal acoustic rhythms do not reflect size, morphology, social structure (local population size), environment, etc., but do they necessarily support the hypothesis that a fundamental CNS property underlies these rhythm values ?   That is, delta waves (or their equivalents) are well known from humans, but how widespread are they in mammals, other vertebrates, and beyond?  And if such CNS waves are found widely, do they always have the same frequency ? 

We thank the reviewer for pointing out the lack of clear information regarding the presence and range of delta oscillations across taxa. Delta oscillations are indeed observed in most major animal groups. They have been well described in several mammalian species (1–4 Hz; Buzsáki et al., 2023), in reptiles such as turtles and geckos where EEG activity is dominated by a single spiking rhythm around 2 Hz (Knyazev, 2012), and even in insects, where delta-range oscillations (1–3 Hz) have been reported during sleep in mosquitoes (Raccuglia et al., 2019). These references and clarifications have been added to the Discussion (lines 200-206).

At the bottom of page 2 the authors note that environmental constraints may affect animal acoustic rhythms ; i.e. the communication message has to arrive relatively intact at the receiver, who may be rather distant.  Here, you are referring to the phenomenon of reverberation wherein the fine temporal structure (pulsing) of a sound message can be degraded in a complex environment (dense vegetation, for example).  This phenomenon is not due so much to the rhythm rate (e.g. pulses per second) as to the duty cycle (pulse duration / pulse period).  That is, when duty cycle is high, degradation (reverberation effects) is likely to be much greater.  Can this detail be discussed and factored in your analysis of the potential for environmental features influencing rhythm?  (In fact, some work has already been done on this issue in insects and birds.)

We thank the reviewer for this valuable clarification and have now addressed this point in the revised discussion (lines 189-191), where we explicitly acknowledge the limitation of our environmental predictors and discuss how factors such as duty cycle and reverberation could further influence acoustic rhythm across habitats.

Communication is not just the sender, but also the receiver !  Animal signals often incorporate a considerable amount of complexity in their temporal structure, but much of that structure may be an incidental byproduct of the way sounds are produced and have little effect on the receiver's perception and response.  I do not see this critical point being considered in the study.  The authors have used appropriate analysis to extract the most 'conspicuous' rhythm component from an animal's acoustic sequence, but this most conspicuous component may not be the critical one in communication.  For examples, using two of the three insect species in the list of 98 species sampled, nested rhythms are present.  In the cricket Gryllus bimaculatus, there is a 30-Hz pulse rhythm produced directly by the wingstroke rate during singing, and then there is an approx. 3-Hz chirp rate (which we humans hear and appreciate).  The 30-Hz pulse rhythm is absolutely essential for species
recognition, whereas the 3-Hz chirp rhythm is of much less general importance, and may influence sexual and social selection.  Similarly, in Apis mellifera (Western honeybee), there is a rate of waggling during the dance, and also a rate at which the dance (figure 8 movement) is repeated.  Which one(s) are you going to use?  And, it is not clear how important the acoustic component of the signal is to the receiver honeybee (but perhaps the conserved rhythm applies to other, non-acoustic modalities as well).  Essentially, much more attention to details of communication would be needed for a thorough analysis, even in a broad comparative study.

We thank the reviewer for raising this important point. We could not agree more that communication involves both the sender and the receiver, and that not all temporal features of a signal hold equal communicative value. In this study, we focused specifically on slow amplitude variations, applying a 20 Hz cut-off to compute the acoustic envelope. This choice was motivated by our aim to characterize the slower temporal dynamics that structure acoustic communication. We do not claim that the rhythm observed around 2–3 Hz is the only relevant timescale in animal vocalizations. As the reviewer rightly notes, many species, particularly insects, exhibit nested rhythmic structures. For instance, in cricket species, 30 Hz pulses are grouped into chirps around 3 Hz. The roughness range also characterizes many communication signals, particularly for alerting. In such cases, the fast pulse rate carries species-specific recognition cues. Our claim, which doesn’t invalidate this fact, is that the slower amplitude modulations correspond to the timescale at which neural oscillations best integrate auditory information. We also acknowledge that the interaction between these slow and fast timescales remains an open and promising question, as noted in the discussion (lines 199-225).

Phylogenetic sampling.  The 98 species represent a broad sampling of birds, and some mammals and a few anuran amphibians.  What is the potential for some bias influencing which species made the list and which did not ?  Can this be evaluated for birds at least, where the coverage is best ? Given the extensive acoustic data in the Xeno Canto library, would it be possible, and preferable, to conduct an in-depth analysis of several taxa (families?) in order to avoid the sampling bias problem, and also the problem of phylogenetic analysis on incomplete data sets ?  You would also be afforded more accurate data on morphology, behavior, environment, etc. than is possible with the approach used in the current manuscript (all acoustic taxa ; sampled species widely scattered and poorly sampled, save for birds).

We thank the reviewer for this constructive comment. Our goal was to perform a broad comparative analysis across tetrapods, rather than to conduct an in-depth study of particular families or genera. For this reason, our sampling strategy was designed to reflect the natural distribution of taxonomic diversity rather than to artificially balance the number of species per clade. Consequently, our dataset includes 58 bird species and 28 mammal species, which roughly mirrors the actual proportion of species richness between these two groups.

We acknowledge that coverage of other tetrapod orders is more limited, and we have now focused our main analyses on birds and mammals, where data availability and recording quality are highest. These two clades yielded highly consistent results, with a conserved rhythm of acoustic signals in the delta range (mean rhythm = 2.73 Hz; 95% HDI [0.45–4.99]). When analyzed separately, both groups showed similar tendencies (Mammals: 2.3 Hz, 95% HDI [0.34–4.8]; Birds: 2.8 Hz, 95% HDI [0.34–5.6]), confirming that the relationship is not driven by the overrepresentation of a single clade but reflects a shared pattern across independent lineages.

We also emphasize that our aim was to identify macroevolutionary trends rather than to test the influence of ecological, morphological, or behavioral factors at a microevolutionary scale. While we do not exclude the contribution of such factors to rhythm variation within specific taxa, their effects are expected to produce local fluctuations rather than consistent evolutionary constraints. The comparative phylogenetic analysis supports this view: the evolutionary dynamics of rhythm follow an Ornstein–Uhlenbeck (OU) process, with 93% of phylogenetic half-lives below one, indicating strong stabilizing selection toward a common optimum. If rhythm were primarily shaped by extrinsic or lineage-specific factors, we would instead expect a Brownian motion process, reflecting gradual, unconstrained divergence. The OU pattern therefore suggests the existence of a deeply conserved, endogenous constraint acting across lineages. We have clarified this interpretation in the revised Discussion (lines 180-198), stating that while our approach cannot exclude the influence of local, short-term factors at the microevolutionary level, the phylogenetic pattern itself effectively rules out their dominance at the macroevolutionary scale.
