# Supplementary material for: Animal acoustic communication has a conserved optimal rhythm within the neural delta range
Source: PLoS Biol. 2026 Jun 9;24(6):e3003798. doi: 10.1371/journal.pbio.3003798 (PMC13249164; doi:10.1371/journal.pbio.3003798)
Supplement: Attachment — Submitted filename: Response_reviewers_R2.docx [file pbio.3003798.s011.docx]

Reviewer #2:

The manuscript has been significantly improved. Information added about how recordings were selected cleared my question before. The authors have also focused on birds and mammals for the phylogenetic history analysis and so most of my earlier questions and criticisms about phylogenetic representation and different mechanisms of rhythmic generation and detection in invertebrates and vertebrates no longer apply. The main conclusion remains the same and stands stronger now.

We appreciate this positive feedback and the recognition that the additional methodological clarifications and the focused phylogenetic analyses have strengthened the study and addressed the concerns raised in the previous round.

However, the issues that those hypotheses species specific selective pressures are vertebrate biased continue to exist. I agree that it is important to include animals other than birds and mammals in the survey of the acoustic data to show that a low rhythm is widespread among acoustically communicating animals. I also think focusing on birds and mammals only for the phylogenetic pattern is a wise choice. Doing so does not undermine how novel and exciting the new hypothesis is and make the possibility sounder and more rigorous. Along the same line, I suggest the authors to run the Bayesian model using only birds and mammals and move model output with everything included to the supplementary. Many mechanisms the authors mention in the Introduction, such as relationship with articulators inherited from mastication, breathing rate, and social complexity apply to vertebrates and vocally communicating animals. Such mechanisms do not apply to animals that produce sound by, for example, rubbing wings or vibrating substrates with legs, for which sounds production does not rely on air exchange. In addition, data about social complexity is highly biased towards eusocial vertebrates and for many other taxa, it is difficult to distinguish whether there is lack of social complexity or lack of data. New data from what we used to think as simple, non-social animals keep surprising us. Although the model itself is robust to low sample size in some groups, the rationale to include them in the first place is not as sound. By using a two-tiered presentation of the results, those with sound mechanistic rationale and rich and high-quality data in the main results and the rest in the supplementary to show the potential of the model, the author can support their point on a much more solid ground. Otherwise, anyone who studies acoustic communication in insects may have problem with the rationales in the Introduction. Because the idea about delta oscillation and signal reception is novel enough, whether they apply to insects or not does not make it or break it. Even if the mechanism turns out to be not true in, for example, fish and insects, I don’t think this makes the idea any less exciting.

As suggested, we have implemented a two-tiered presentation of the results, reporting in the main manuscript a bayesian model restricted to birds and mammals. The latter yields consistent results with those obtained using the full multi-taxon dataset and further strengthens them, as the weak interaction between mastication and weight is no longer observed. The full model has been moved to the supplementary information, serving as an exploratory approach.

Reviewer #3: This revised version has addressed many of the points raised by referees, but some critical issues remain and should be clarified / corrected before publication.

A minor point that arises in the beginning of the manuscript is that the authors refer to theta waves (in the brain) on several occasions, and then switch to focus on delta waves for the remainder of the paper.  My understanding is that these two categories of waves differ in frequency, and possibly in function.  Please clarify.

Theta and delta oscillations indeed differ in frequency range and have been associated with partially distinct functional roles. While the reference framework of this study is the role of the theta-band rhythm (4–8 Hz) in human speech processing, delta oscillations (1-4 Hz) also play a role in this process. We have now added this information to the introduction lines (59-60) . Interestingly, our comparative analyses revealed that the dominant rhythm across species clusters around ~3 Hz, which falls within the delta range, rather than the more dominant human theta rhythm and which is why our discussion then focuses more on this band.

I strongly suggest that the authors restrict their analysis / treatment to birds, and then (secondarily) mammals, and forgo including all other taxa in formal analysis - including supplementary material.  The number of species sampled in these other groups is far too small, and their behavior is so different from that of avian and mammalian rhythm that they cannot be considered together (apples and oranges).  You simply cannot defend the attempt to represent a taxon such as Insecta with four species.

This is an important point also raised by the other reviewer. We now present an analysis including only birds and mammals, which yields similar results as the full analysis. The latter is now only presented in supplemental material, serving as an exploratory approach indicating a possible generalization (and we hope spark further investigation).

nota bien : If insects are included, data from many species would clearly refute the delta wave hypothesis, as the receivers (eg. females evaluating male calling songs) typically evaluate high-frquency pulse rates and pay little attention to low frequency (2-4 Hz) chirp rates.

We agree that, in many species, critical information is conveyed at faster temporal rates, often in the low-and high gamma range. This is well documented in insects, where receivers such as females evaluating male calling songs rely on rapid pulse rates. A similar principle applies to human speech, in which phonemic information is carried at low-gamma timescales (~30 Hz).

However, the presence of fast information-bearing modulations does not imply the irrelevance of slower rhythmic structure. In humans, for example, the intelligibility of time-compressed speech can be substantially restored by reintroducing slow suprasegmental temporal structure (e.g., by inserting silences aligned with prosodic boundaries). In such cases, information remains encoded at fast timescales but becomes perceptually accessible only when embedded within an appropriate slower rhythmic framework [(*1*)](https://www.zotero.org/google-docs/?0gbUXp).

Our claim is therefore not that species “encode” information in the delta range but that this slow rhythm might be a common channel for signal parsing, attentional alignment, or perceptual grouping, whereas the fine-grained content is conveyed via faster modulations. Our framework is based on a two-timescale model of acoustic processing, integrating both slow and fast temporal dynamics.

Continuing, it may be best to concentrate mostly on birds, where vocalization is nearly universal and used in intra-specific communication;  see next point.

Following the (authors' ) point that the delta-wave hypothesis focuses on the receiver as opposed to the signaler, the calls should be restricted to 'songs' used in intra-specific communication, being careful to avoid including alarm and startle vocalizations. These functions should be readily obtained from Xeno Canto and other main references.

Given the depth of song records in Xeno Canto for many, many bird species of the world, I found it curious that the authors restricted their sampling to so few species (68) even when accounting for their several stipulations (quality of recording, balanced phylogenetic representation, etc.).  More species sampling could only strengthen the study.

If the study focuses mostly on birds, there should be no need for creating you own MtDNA phylogeny, as rather thorough avian phylogeny is now available.

We agree that birds represent a particularly informative clade for the study of acoustic communication, given both the near universality of vocal signaling and the exceptional depth of available recordings (e.g., Xeno Canto). We also acknowledge that our representation of some other clades (e.g., insects, reptiles, fish) is more limited. In response to this concern, we have moved these groups to the supplementary analyses, where they serve an exploratory and comparative purpose rather than forming the core of the phylogenetic inference.

However, the central aim of our work is to test whether shared rhythmic constraints emerge across phylogenetically distant taxa, rather than within a single clade. For this reason, we did not restrict our analyses to birds, nor to a single vocalization category such as intra-specific “songs.” Our framework is receiver-centered and focuses on temporal processing constraints that may apply broadly to biologically relevant acoustic signals.

Testing the delta wave hypothesis :  You might look at outliers in song rhythm among species, and avoid considering that all species have the same delta wave frequency.  Do species with faster song rhythms, such as those with significant trill elements that receivers do focus on, exhibit higher delta wave frequencies ?  This information may not be available at present, but it is the sort of data that could lend itself to more objective testing of the hypothesis.  A 'future directions' section at the end of the manuscript would be appropriate.

This is indeed a very interesting suggestion for a follow-up study. Exploring rhythmic outliers and the interaction between the slow temporal structure of communication signals with their fast fine-structure (e.g. trill) is an important research direction, which we now mention in the discussion lines 253-255.

References:

[1.](https://www.zotero.org/google-docs/?WdvfCc)	[O. Ghitza, S. Greenberg, On the Possible Role of Brain Rhythms in Speech Perception: Intelligibility of Time-Compressed Speech with Periodic and Aperiodic Insertions of Silence. *Phonetica* **66**, 113–126 (2009).](https://www.zotero.org/google-docs/?WdvfCc)
