# Supplementary material for: Animal acoustic communication has a conserved optimal rhythm within the neural delta range
Source: PLoS Biol. 2026 Jun 9;24(6):e3003798. doi: 10.1371/journal.pbio.3003798 (PMC13249164; doi:10.1371/journal.pbio.3003798)
Supplement: Peer review history

# Editor report: Decision Letter 0

**DOI:** 10.1371/journal.pbio.3003798.r001

**Authors:** Roland Roberts

18 Jul 2025

Dear Dr Piette,

Thank you for submitting your manuscript entitled "A conserved rhythm in animal acoustic communication" for consideration as a Research Article by PLOS Biology.

Your manuscript has now been evaluated by the PLOS Biology editorial staff and I’m writing to let you know that we would like to send your submission out for external peer review.

However, before we can send your manuscript to reviewers, we need you to complete your submission by providing the metadata that is required for full assessment. To this end, please login to Editorial Manager where you will find the paper in the ‘Submissions Needing Revisions’ folder on your homepage. Please click ‘Revise Submission’ from the Action Links and complete all additional questions in the submission questionnaire.

Once your full submission is complete, your paper will undergo a series of checks in preparation for peer review. After your manuscript has passed the checks it will be sent out for review. To provide the metadata for your submission, please Login to Editorial Manager (https://www.editorialmanager.com/pbiology) within two working days, i.e. by Jul 22 2025 11:59PM.

If your manuscript has been previously peer-reviewed at another journal, PLOS Biology is willing to work with those reviews in order to avoid re-starting the process. Submission of the previous reviews is entirely optional and our ability to use them effectively will depend on the willingness of the previous journal to confirm the content of the reports and share the reviewer identities. Please note that we reserve the right to invite additional reviewers if we consider that additional/independent reviewers are needed, although we aim to avoid this as far as possible. In our experience, working with previous reviews does save time.

If you would like us to consider previous reviewer reports, please edit your cover letter to let us know and include the name of the journal where the work was previously considered and the manuscript ID it was given. In addition, please upload a response to the reviews as a 'Prior Peer Review' file type, which should include the reports in full and a point-by-point reply detailing how you have or plan to address the reviewers' concerns.

During the process of completing your manuscript submission, you will be invited to opt-in to posting your pre-review manuscript as a bioRxiv preprint. Visit http://journals.plos.org/plosbiology/s/preprints for full details. If you consent to posting your current manuscript as a preprint, please upload a single Preprint PDF.

Feel free to email us at plosbiology@plos.org if you have any queries relating to your submission.

Kind regards,

Roli Roberts

Roland Roberts, PhD

Senior Editor

PLOS Biology

rroberts@plos.org

# Peer review: Decision Letter 1

**DOI:** 10.1371/journal.pbio.3003798.r002

**Authors:** Roland Roberts

19 Sep 2025

Dear Dr Piette,

Thank you for your patience while your manuscript "A conserved rhythm in animal acoustic communication" was peer-reviewed at PLOS Biology. It has now been evaluated by the PLOS Biology editors, an Academic Editor with relevant expertise, and by three independent reviewers.

You'll see that reviewer #1 only has one minor request. Reviewer #2 is broadly positive about the study, but has some significant concerns around the small numbers and taxonomic bias; there is also a lack of methodological clarity to ensure against cherry-picking, and s/he has a number of other issues about the model. S/he wonders if focussing on birds and mammals might strengthen the paper, and is rather sceptical that you can exclude three of your four hypotheses so confidently. Reviewer #3 is also positive, but again is sceptical that your hypothesis is well supported. S/he raises a number of other points, including issues of bias and sampling (suggesting the Xeno Canto library).

In light of the reviews, which you will find at the end of this email, we would like to invite you to revise the work to thoroughly address the reviewers' reports.

Given the extent of revision needed, we cannot make a decision about publication until we have seen the revised manuscript and your response to the reviewers' comments. Your revised manuscript is likely to be sent for further evaluation by all or a subset of the reviewers.

In addition to these revisions, you will need to complete some formatting changes, which you will receive in a follow up email. A member of our team will be in touch with a set of requests shortly.

We expect to receive your revised manuscript within 3 months. Please email us (plosbiology@plos.org) if you have any questions or concerns, or would like to request an extension.

At this stage, your manuscript remains formally under active consideration at our journal; please notify us by email if you do not intend to submit a revision so that we may withdraw it.

**IMPORTANT - SUBMITTING YOUR REVISION**

Your revisions should address the specific points made by each reviewer. Please submit the following files along with your revised manuscript:

1. A 'Response to Reviewers' file - this should detail your responses to the editorial requests, present a point-by-point response to all of the reviewers' comments, and indicate the changes made to the manuscript.

*NOTE: In your point-by-point response to the reviewers, please provide the full context of each review. Do not selectively quote paragraphs or sentences to reply to. The entire set of reviewer comments should be present in full and each specific point should be responded to individually, point by point.

You should also cite any additional relevant literature that has been published since the original submission and mention any additional citations in your response.

2. In addition to a clean copy of the manuscript, please also upload a 'track-changes' version of your manuscript that specifies the edits made. This should be uploaded as a "Revised Article with Changes Highlighted" file type.

*Re-submission Checklist*

When you are ready to resubmit your revised manuscript, please refer to this re-submission checklist: https://plos.io/Biology_Checklist

To submit a revised version of your manuscript, please go to https://www.editorialmanager.com/pbiology/ and log in as an Author. Click the link labelled 'Submissions Needing Revision' where you will find your submission record.

Please make sure to read the following important policies and guidelines while preparing your revision:

*Published Peer Review*

Please note while forming your response, if your article is accepted, you may have the opportunity to make the peer review history publicly available. The record will include editor decision letters (with reviews) and your responses to reviewer comments. If eligible, we will contact you to opt in or out. Please see here for more details:

https://blogs.plos.org/plos/2019/05/plos-journals-now-open-for-published-peer-review/

*PLOS Data Policy*

Please note that as a condition of publication PLOS' data policy (http://journals.plos.org/plosbiology/s/data-availability) requires that you make available all data used to draw the conclusions arrived at in your manuscript. If you have not already done so, you must include any data used in your manuscript either in appropriate repositories, within the body of the manuscript, or as supporting information (N.B. this includes any numerical values that were used to generate graphs, histograms etc.). For an example see here: http://www.plosbiology.org/article/info%3Adoi%2F10.1371%2Fjournal.pbio.1001908#s5

*Protocols deposition*

To enhance the reproducibility of your results, we recommend that if applicable you deposit your laboratory protocols in protocols.io, where a protocol can be assigned its own identifier (DOI) such that it can be cited independently in the future. Additionally, PLOS ONE offers an option for publishing peer-reviewed Lab Protocol articles, which describe protocols hosted on protocols.io. Read more information on sharing protocols at https://plos.org/protocols?utm_medium=editorial-email&utm_source=authorletters&utm_campaign=protocols

Thank you again for your submission to our journal. We hope that our editorial process has been constructive thus far, and we welcome your feedback at any time. Please don't hesitate to contact us if you have any questions or comments.

Sincerely,

Roli Roberts

Roland Roberts, PhD

Senior Editor

PLOS Biology

rroberts@plos.org

------------------------------------

REVIEWERS' COMMENTS:

Reviewer #1:

This is a very good Discovery Report, clearly presented, with the right amount of data backing up the main claim, and the right level of Discussion. I am happy to recommend publication.

Concerning the Discussion, I was wondering if the authors would find the following findings noteworth in the context of their Discussion (https://www.biorxiv.org/content/10.1101/2024.12.02.626521v1)

Reviewer #2:

This manuscript presents an observation of an overrepresentation of temporal tempo below 5 Hz in acoustic signals across animal phyla and subsequently tests four non-mutually exclusive hypotheses for the evolution of the observed pattern. These hypotheses include both ultimate explanations (signal efficacy, i.e., transmission in the environment and social function) and proximate explanations (neural mechanism and physiological constraints).

The questions asked in this manuscript are both intriguing and novel and the scope of interest is wide. Temporal structures are a ubiquitous feature of acoustic signals of animals. The question whether temporal rhythm is distributed randomly or does its distribution show some pattern across animals is an intriguing and novel one. If a pattern is observed, asking what gives rise to the pattern should be of interest for a wide range of fields, including neuroscience, animal behavior, and evolutionary biology. Among the four hypotheses tested, the latter three (physiological constraints, environmental influence, and social influence) are not new. These hypotheses have been proposal and tested in similar contexts. The first hypothesis about a deeply conserved neural mechanism is novel. I applaud the authors' effort to try to bring together neurobiology, behavior, and evolution together. Such a perspective may foster cross talk among fields that do not talk to each other enough and has the potential to generate transformative insights.

The idea about rhythmicity in acoustic signals and delta oscillation in the brain is bold. This inherently means the idea comes with some degree of forward-projecting, with more gaps in the current evidence chain than some other less bold ideas. I believe these alone should not be the reason to kill bold ideas. The key lies in whether our current theory and empirical data points to a promising path for further investigation. With all these in mind, this manuscript falls short of demonstrating careful and thorough examination of existing theory and data and construction of a solid foundation for the novel idea.

The conclusion that there is an overrepresentation of optimum rhythmic tempo in acoustic signals below 5 Hz across animals lack validity. First, this conclusion come from too low a sample size. Specifically, besides birds and mammals, the other classes are represented by 4 or even only 1 species. Even the number of bird and mammal species are not very high. Such a sample size is extremely biased in terms of taxonomic group and it is difficult to imagine this small sample can truthfully reflect the diversity in taxonomic groups and temporal structures in acoustic signals. Second, there is no detailed description about how these species and acoustic samples are chosen. It is thus impossible to determine whether the samples are chosen with a reasonable criteria. The fact that exceptions are plentiful, but the selected data show a very strong pattern raises the possibility of cherry-picking. For example, in insects, the repetitive elements in the song could occur at high rates, some are a magnitude higher (Grobe et al., 2012; Skovmand and Boel Pedersen, 1983; Spooner, 1964; Ulagaraj, 1976; Ulagaraj and Walker, 1975; von Schilcher, 1976; Walker, 2000). What are the reasons the one insect selected falls within the so-called optimum tempo range but these species whose tempo falls outside the range were not included? Third, acoustic sample were collected from multiple sources, including some online video platforms where it is difficult to standardize the recording condition and quality. For example, if the acoustic samples were recorded in an environment where there were noise sources that show rhythmic pattern, how can the focal signal and these noises be teased apart? In ectotherm animals, it is well-known that the temporal rhythm of the acoustic signal change with ambient temperature. For endotherm animals, the body temperature may vary across species and the temporal pattern of the acoustic signals may also vary with body temperature. How is temperature standardized across samples? Because the models cannot outperform input data, the conclusions from models with problematic input data are thus unsound. And as this conclusion forms the foundation of the hypotheses tested, the entire study is built on a shaky ground.

Going forward, there can be a few options. My suggestion is to focus on birds and mammals only for a few reasons. First, the most novel hypothesis about delta oscillation are best fit for birds and mammals (see detailed reasons below in my comments about this hypothesis). Second, with the magnitude of taxonomic diversity of invertebrates and especially insects, it is an extremely difficult task to collect sound samples and data for environment, social complexity and both parameters that can be representative of this group. If the authors focus on just birds and mammals, it is much more manageable. Next, I suggest the authors to:

(1) Come up with universal and standardized protocol and criteria for data search and filter. There needs to be a standardized protocol for systematically searching the literature and databases. This protocol should specifically address both coverage of the search and measures to mitigate bias in data inclusion. There also should be universal criteria for selecting and processing sound samples.

(2) Increase sample size such that the sample size is sufficient to represent the taxonomic group the authors intend to focus on

Second, I think the hypothesis about delta oscillation playing a role in the generation of a certain rhythmic tempo is very intriguing and bold but I have several concerns about this hypothesis. First, the range of taxonomic group the authors proposed may be too wide. The neural mechanism for the generation of rhythmic pattern in songs of insects, frogs and fish have been well studied. In all of these animals, the central pattern generator is a major driver of the rhythmic pattern (Bass, 2014; Clyne and Miesenböck, 2008; Grobe et al., 2012; Rhodes et al., 2007; Schöneich and Hedwig, 2011, 2017; Skovmand and Boel Pedersen, 1983; Spooner, 1964; Ulagaraj, 1976; Ulagaraj and Walker, 1975; von Schilcher, 1976; Walker, 2000). I may be wrong but so far, I have not found any evidence that delta oscillation plays any role in the output of central pattern generators besides potential entrain or modulation in these animals. This raises the question about whether the proposed mechanism apply to these taxonomic groups or not. If there are good evidence showing that the delta rhythm determines the output of singing CPG in these animals, the authors should add the evidence to the manuscript. Second, just because the tempo of two phenomena coincide does not necessarily imply causality. To establish causality, I expect a more solid logic chain supported by current theory and empirical data. The links on this chain may not be all connected yet but all (or at least most) steps of this logic chain needs to be clearly presented to make this hypothesis more plausible. Because behavioral and neural organizations represent different levels of organizations that can be independent of each other (Katz, 2016), it is entirely possible these two things have nothing to do with each other or the relationship is not what appears on the surface. And a minor point, when the hypothesis was first presented, the authors mentioned theta, instead of delta, rhythm. This however, does not mean there is no wisdom to the idea. I suggest the authors to present a more concrete mechanism that can be backed up by theory and data.

Another major problem is that support for this hypothesis is through process of elimination and there is no actual test of this hypothesis. This significantly weakens the validity of the conclusion because the four hypotheses are not exhaustive and the tests of the other hypothesis are problematic such that they cannot be confidently rejected (see comments below).

The other hypotheses and their tests have various problem as well. First, the hypothesis about morphological and physiological constraints is very vertebrate-centric. It was not explicitly stated but I'm guessing that weight and mastication status (and it seems beak morphology for birds too) are the two factors used to test this hypothesis. Invertebrates produce sounds by entirely different mechanisms such that these factors do not apply to them. Even for vertebrates, the constraints can be trade-offs in vocal performance that these factors cannot capture. For example, in birds, it is well known that there is a constraint in vocal performance such that there are tradeoffs between trill rate and frequency band width and learning accuracy (Ballentine et al., 2004; Podos et al., 2004).

The hypothesis about environmental constraints, or signal efficacy is valid but the citation the authors cited (citation 18) is not a good one. Please cite a better source. There are many papers from behavioral ecology on this topic. However, data used for testing the hypothesis is crude and the accuracy questionable as these data mainly come from two books that were not peer reviewed. It is unclear how the authors of these books collected these data and if multiple sources exist, how were data selected. This criticism applies to tests of all three hypotheses except for the first one.

Last, I fail to understand the rationale for the hypothesis about rhythm and social complexity. Social complexity can affect vocal repertoire but how does this inform us about the relationship between social complexity and temporal rhythm? On the other hand, social interactions may very well influence signal design, including temporal rhythm. For example, repetition rate may increase in aggressive contexts. In the context of mate choice, females may prefer either a specific rate or fast rate. Males competing to gain access to mate are selected to perform according to what females prefer. Such influences may be taxonomic- and context-dependent and I'm not sure how a universal factor can capture these relationships.

Due to these reasons, the conclusion that these three hypotheses should be rejected is not sound. I suggest the authors to carefully reconsider the hypotheses to make sure they are reasonable and are well backed up by theory and empirical data. Testing these hypotheses, however, may be a much bigger task than what was done in this study. It is doable but a good test needs to be built on good quality data, meticulous scrutiny and curation of the collected data, as well as finding ways to come up with parameter that can both capture the relationship and are universal among all animals tested.

Ballentine, B., J. Hyman, and S. Nowicki. 2004. Vocal performance influences female response to male bird song: an experimental test. Behavioral Ecology, 15:163-168.

Bass, A. H. 2014. Central pattern generator for vocalization: is there a vertebrate morphotype? Current opinion in neurobiology, 28:94-100.

Clyne, J. D., and G. Miesenböck. 2008. Sex-specific control and tuning of the pattern generator for courtship song in Drosophila. Cell, 133:354-363.

Grobe, B., M. M. Rothbart, A. Hanschke, and R. M. Hennig. 2012. Auditory processing at two time scales by the cricket Gryllus bimaculatus. Journal of Experimental Biology, 215:1681-1690.

Katz, P. S. 2016. Evolution of central pattern generators and rhythmic behaviours. Phil. Trans. R. Soc. B, 371:20150057.

Podos, J., S. K. Huber, and B. Taft. 2004. Bird song: the interface of evolution and mechanism. Annu. Rev. Ecol. Evol. Syst., 35:55-87.

Rhodes, H. J., J. Y. Heather, and A. Yamaguchi. 2007. Xenopus vocalizations are controlled by a sexually differentiated hindbrain central pattern generator. Journal of Neuroscience, 27:1485-1497.

Schöneich, S., and B. Hedwig. 2011. Neural basis of singing in crickets: central pattern generation in abdominal ganglia. Naturwissenschaften, 98:1069-1073.

—. 2017. Neurons and Networks Underlying Singing Behaviour, The Cricket as a Model Organism, pp. 141-153: Springer.

Skovmand, O., and S. Boel Pedersen. 1983. Song recognition and song pattern in a shorthorned grasshopper. Journal of comparative physiology, 153:393-401.

Spooner, J. D. 1964. The Texas bush katydid—its sounds and their significance. Animal Behaviour, 12:235-IN4.

Ulagaraj, S. 1976. Sound production in mole crickets (Orthoptera: Gryllotalpidae: Scapteriscus). Annals of the Entomological Society of America, 69:299-306.

Ulagaraj, S., and T. J. Walker. 1975. Response of flying mole crickets to three parameters of synthetic songs broadcast outdoors. Nature, 253:530-532.

von Schilcher, F. 1976. The function of pulse song and sine song in the courtship of Drosophila melanogaster. Animal Behaviour, 24:622-625.

Walker, T. J. 2000. Pulse rates in the songs of trilling field crickets (Orthoptera: Gryllidae: Gryllus). Annals of the Entomological Society of America, 93:565-572.

Reviewer #3:

Review of 'A conserved rhythm in animal acoustic communication' (PBIOLOGY-D-25-02159R1)

This manuscript addresses a current issue in animal behavior, cognition and neuroscience, and could potentially be of interest to many readers. For the most part, the authors have carefully followed protocols for the comparative and phylogenetic method, have explained their quantitative analyses and assumptions, and claim that animal rhythms - in acoustic communication (including human speech and music) - generally fall within a narrow tempo (repetition rate) range and may do so because of fundamental properties of central nervous systems. Below, I offer recommendations that could sharpen the analyses, and I also note some caveats.

The authors present a body of results that may support the hypothesis that the typical values of animal acoustic rhythms do not reflect size, morphology, social structure (local population size), environment, etc., but do they necessarily support the hypothesis that a fundamental CNS property underlies these rhythm values ? That is, delta waves (or their equivalents) are well known from humans, but how widespread are they in mammals, other vertebrates, and beyond? And if such CNS waves are found widely, do they always have the same frequency ?

At the bottom of page 2 the authors note that environmental constraints may affect animal acoustic rhythms ; i.e. the communication message has to arrive relatively intact at the receiver, who may be rather distant. Here, you are referring to the phenomenon of reverberation wherein the fine temporal structure (pulsing) of a sound message can be degraded in a complex environment (dense vegetation, for example). This phenomenon is not due so much to the rhythm rate (e.g. pulses per second) as to the duty cycle (pulse duration / pulse period). That is, when duty cycle is high, degradation (reverberation effects) is likely to be much greater. Can this detail be discussed and factored in your analysis of the potential for environmental features influencing rhythm? (In fact, some work has already been done on this issue in insects and birds.)

Communication is not just the sender, but also the receiver ! Animal signals often incorporate a considerable amount of complexity in their temporal structure, but much of that structure may be an incidental byproduct of the way sounds are produced and have little effect on the receiver's perception and response. I do not see this critical point being considered in the study. The authors have used appropriate analysis to extract the most 'conspicuous' rhythm component from an animal's acoustic sequence, but this most conspicuous component may not be the critical one in communication. For examples, using two of the three insect species in the list of 98 species sampled, nested rhythms are present. In the cricket Gryllus bimaculatus, there is a 30-Hz pulse rhythm produced directly by the wingstroke rate during singing, and then there is an approx. 3-Hz chirp rate (which we humans hear and appreciate). The 30-Hz pulse rhythm is absolutely essential for species recognition, whereas the 3-Hz chirp rhythm is of much less general importance, and may influence sexual and social selection. Similarly, in Apis mellifera (Western honeybee), there is a rate of waggling during the dance, and also a rate at which the dance (figure 8 movement) is repeated. Which one(s) are you going to use? And, it is not clear how important the acoustic component of the signal is to the receiver honeybee (but perhaps the conserved rhythm applies to other, non-acoustic modalities as well). Essentially, much more attention to details of communication would be needed for a thorough analysis, even in a broad comparative study.

Phylogenetic sampling. The 98 species represent a broad sampling of birds, and some mammals and a few anuran amphibians. What is the potential for some bias influencing which species made the list and which did not ? Can this be evaluated for birds at least, where the coverage is best ?

Given the extensive acoustic data in the Xeno Canto library, would it be possible, and preferable, to conduct an in-depth analysis of several taxa (families?) in order to avoid the sampling bias problem, and also the problem of phylogenetic analysis on incomplete data sets ? You would also be afforded more accurate data on mophology, behavior, environment, etc. than is possible with the approach used in the current manuscript (all acoustic taxa ; sampled species widely scattered and poorly sampled, save for birds).

# Author comment: Author response to Decision Letter 2

**DOI:** 10.1371/journal.pbio.3003798.r003

13 Jan 2026

Attachment Submitted filename: Response_Reviewver_Plos_Biology.docx

# Peer review: Decision Letter 2

**DOI:** 10.1371/journal.pbio.3003798.r004

**Authors:** Roland Roberts

21 Feb 2026

Dear Dr Piette,

Thank you for your patience while we considered your revised manuscript "A conserved rhythm in animal acoustic communication" for consideration as a Discovery Report at PLOS Biology. Your revised study has now been evaluated by the PLOS Biology editors, the Academic Editor, and two of the original reviewers.

In light of the reviews, which you will find at the end of this email, we are pleased to offer you the opportunity to address the remaining points from the reviewers in a revision that we anticipate should not take you very long. We will then assess your revised manuscript and your response to the reviewers' comments with our Academic Editor aiming to avoid further rounds of peer-review, although we might need to consult with the reviewers, depending on the nature of the revisions.

You'll see that reviewer #2 says that the manuscript has been significantly improved, but suggests that you also run the Bayesian model with just mammals and birds, focussing the main paper only on these better supported aspects and presenting the remainder in the SI. Reviewer #3 makes a similar point, even going as far as to prioritise birds over mammals, and focussing on intra-specific calls.

In addition to these revisions, you may need to complete some formatting changes, which you will receive in a follow up email. A member of our team will be in touch with a set of requests shortly. If you do not receive a separate email within a few days, please assume that checks have been completed, and no additional changes are required.

We expect to receive your revised manuscript within 1 month. Please email us (plosbiology@plos.org) if you have any questions or concerns, or would like to request an extension.

At this stage, your manuscript remains formally under active consideration at our journal; please notify us by email if you do not intend to submit a revision so that we withdraw the manuscript.

**IMPORTANT - SUBMITTING YOUR REVISION**

Your revisions should address the specific points made by each reviewer. Please submit the following files along with your revised manuscript:

1. A 'Response to Reviewers' file - this should detail your responses to the editorial requests, present a point-by-point response to all of the reviewers' comments, and indicate the changes made to the manuscript.

*NOTE: In your point-by-point response to the reviewers, please provide the full context of each review. Do not selectively quote paragraphs or sentences to reply to. The entire set of reviewer comments should be present in full and each specific point should be responded to individually.

You should also cite any additional relevant literature that has been published since the original submission and mention any additional citations in your response.

2. In addition to a clean copy of the manuscript, please also upload a 'track-changes' version of your manuscript that specifies the edits made. This should be uploaded as a "Revised Article with Changes Highlighted " file type.

*Resubmission Checklist*

When you are ready to resubmit your revised manuscript, please refer to this resubmission checklist: https://plos.io/Biology_Checklist

To submit a revised version of your manuscript, please go to https://www.editorialmanager.com/pbiology/ and log in as an Author. Click the link labelled 'Submissions Needing Revision' where you will find your submission record.

Please make sure to read the following important policies and guidelines while preparing your revision:

*Published Peer Review*

Please note while forming your response, if your article is accepted, you may have the opportunity to make the peer review history publicly available. The record will include editor decision letters (with reviews) and your responses to reviewer comments. If eligible, we will contact you to opt in or out. Please see here for more details:

https://blogs.plos.org/plos/2019/05/plos-journals-now-open-for-published-peer-review/

*PLOS Data Policy*

Please note that as a condition of publication PLOS' data policy (http://journals.plos.org/plosbiology/s/data-availability) requires that you make available all data used to draw the conclusions arrived at in your manuscript. If you have not already done so, you must include any data used in your manuscript either in appropriate repositories, within the body of the manuscript, or as supporting information (N.B. this includes any numerical values that were used to generate graphs, histograms etc.). For an example see here: http://www.plosbiology.org/article/info%3Adoi%2F10.1371%2Fjournal.pbio.1001908#s5

*Protocols deposition*

To enhance the reproducibility of your results, we recommend that if applicable you deposit your laboratory protocols in protocols.io, where a protocol can be assigned its own identifier (DOI) such that it can be cited independently in the future. Additionally, PLOS ONE offers an option for publishing peer-reviewed Lab Protocol articles, which describe protocols hosted on protocols.io. Read more information on sharing protocols at https://plos.org/protocols?utm_medium=editorial-email&utm_source=authorletters&utm_campaign=protocols

Thank you again for your submission to our journal. We hope that our editorial process has been constructive thus far, and we welcome your feedback at any time. Please don't hesitate to contact us if you have any questions or comments.

Sincerely,

Roli Roberts

Roland Roberts, PhD

Senior Editor

PLOS Biology

rroberts@plos.org

----------------------------------------------------------------

REVIEWS' COMMENTS:

Reviewer #2:

[see attachment for fully formatted version]

The manuscript has been significantly improved. Information added about how recordings were selected cleared my question before. The authors have also focused on birds and mammals for the phylogenetic history analysis and so most of my earlier questions and criticisms about phylogenetic representation and different mechanisms of rhythmic generation and detection in invertebrates and vertebrates no longer apply. The main conclusion remains the same and stands stronger now.

However, the issues that those hypotheses species specific selective pressures are vertebrate biased continue to exist. I agree that it is important to include animals other than birds and mammals in the survey of the acoustic data to show that a low rhythm is widespread among acoustically communicating animals. I also think focusing on birds and mammals only for the phylogenetic pattern is a wise choice. Doing so does not undermine how novel and exciting the new hypothesis is and make the possibility sounder and more rigorous. Along the same line, I suggest the authors to run the Bayesian model using only birds and mammals and move model output with everything included to the supplementary. Many mechanisms the authors mention in the Introduction, such as relationship with articulators inherited from mastication, breathing rate, and social complexity apply to vertebrates and vocally communicating animals. Such mechanisms do not apply to animals that produce sound by, for example, rubbing wings or vibrating substrates with legs, for which sounds production does not rely on air exchange. In addition, data about social complexity is highly biased towards eusocial vertebrates and for many other taxa, it is difficult to distinguish whether there is lack of social complexity or lack of data. New data from what we used to think as simple, non-social animals keep surprising us. Although the model itself is robust to low sample size in some groups, the rationale to include them in the first place is not as sound. By using a two-tiered presentation of the results, those with sound mechanistic rationale and rich and high-quality data in the main results and the rest in the supplementary to show the potential of the model, the author can support their point on a much more solid ground. Otherwise, anyone who studies acoustic communication in insects may have problem with the rationales in the Introduction. Because the idea about delta oscillation and signal reception is novel enough, whether they apply to insects or not does not make it or break it. Even if the mechanism turns out to be not true in, for example, fish and insects, I don’t think this makes the idea any less exciting.

Reviewer #3: This revised version has addressed many of the points raised by referees, but some critical issues remain and should be clarified / corrected before publication.

A minor point that arises in the beginning of the manuscript is that the authors refer to theta waves (in the brain) on several occasions, and then switch to focus on delta waves for the remainder of the paper. My understanding is that these two categories of waves differ in frequency, and possibly in function. Please clarify.

I strongly suggest that the authors restrict their analysis / treatment to birds, and then (secondarily) mammals, and forgo including all other taxa in formal analysis - including supplementary material. The number of species sampled in these other groups is far too small, and their behavior is so different from that of avian and mammalian rhythm that they cannot be considered together (apples and oranges). You simply cannot defend the attempt to represent a taxon such as Insecta with four species.

nota bien : If insects are included, data from many species would clearly refute the delta wave hypothesis, as the receivers (eg. females evaluating male calling songs) typically evaluate high-frquency pulse rates and pay little attention to low frequency (2-4 Hz) chirp rates.

Continuing, it may be best to concentrate mostly on birds, where vocalization is nearly universal and used in intra-specific communication; see next point.

Following the (authors' ) point that the delta-wave hypothesis focuses on the receiver as opposed to the signaler, the calls should be restricted to 'songs' used in intra-specific communication, being careful to avoid including alarm and startle vocalizations. These functions should be readily obtained from Xeno Canto and other main references.

Given the depth of song records in Xeno Canto for many, many bird species of the world, I found it curious that the authors restricted their sampling to so few species (68) even when accounting for their several stipulations (quality of recording, balanced phylogenetic representation, etc.). More species sampling could only strengthen the study.

If the study focuses mostly on birds, there should be no need for creating you own MtDNA phylogeny, as rather thorough avian phylogeny is now available.

Testing the delta wave hypothesis : You might look at outliers in song rhythm among species, and avoid considering that all species have the same delta wave frequency. Do species with faster song rhythms, such as those with significant trill elements that receivers do focus on, exhibit higher delta wave frequencies ? This information may not be available at present, but it is the sort of data that could lend itself to more objective testing of the hypothesis. A 'future directions' section at the end of the manuscript would be appropriate.

Attachment Submitted filename: Comments for PBIOLOGY-D-25-02159_R2.pdf

# Author comment: Author response to Decision Letter 3

**DOI:** 10.1371/journal.pbio.3003798.r005

10 Apr 2026

Attachment Submitted filename: Response_reviewers_R2.docx

# Editor report: Decision Letter 3

**DOI:** 10.1371/journal.pbio.3003798.r006

**Authors:** Roland Roberts

22 Apr 2026

Dear Theophane,

Thank you for your patience while we considered your revised manuscript "A conserved rhythm in animal acoustic communication" for publication as a Discovery Report at PLOS Biology. This revised version of your manuscript has been evaluated by the PLOS Biology editors and the Academic Editor.

Based on our Academic Editor's assessment of your revision, we are likely to accept this manuscript for publication, provided you satisfactorily address the following data and other policy-related requests.

IMPORTANT - please attend to the following:

a) We need your Title to be more explicitly informative and to contain an active verb. We suggest changing it to "The rhythm of animal acoustic communication has a conserved optimal frequency within the neural delta range"

b) Please address my Data Policy requests below; specifically, we need you to supply the numerical values underlying Figs 1CDE, 2ABCDE, 3ABCDEF, S1AB, S2ABCD, S3AB, S4ABCDE, S5ABCDEFG, S6ABCDEFGHIJ, 7ABCDEF, either as a supplementary data file or as a permanent DOI’d deposition. I note that you already have an associated GitHub deposition (https://github.com/chundrac/phylo-acoustic-rhythm); please could you complete this deposition with the data and code needed to recreate the Figures? Also, because Github depositions can be readily changed or deleted, please make a permanent DOI’d copy (e.g. in Zenodo) and provide this URL (see below).

c) Please cite the location of the data clearly in all relevant main and supplementary Figure legends, e.g. “The data underlying this Figure can be found in S1 Data” or “The data underlying this Figure can be found in https://zenodo.org/records/XXXXXXXX; ; also update your Data Availability Statement accordingly.

d) Please make any custom code available, either as a supplementary file or as part of your data deposition.

e) Please include the URLs of your funders in the Financial Disclosure statement.

As you address these items, please take this last chance to review your reference list to ensure that it is complete and correct. If you have cited papers that have been retracted, please include the rationale for doing so in the manuscript text, or remove these references and replace them with relevant current references. Any changes to the reference list should be mentioned in the cover letter that accompanies your revised manuscript.

In addition to these revisions, you may need to complete some formatting changes, which you will receive in a follow up email. A member of our team will be in touch with a set of requests shortly. If you do not receive a separate email within a few days, please assume that checks have been completed, and no additional changes are required.

We expect to receive your revised manuscript within two weeks.

To submit your revision, please go to https://www.editorialmanager.com/pbiology/ and log in as an Author. Click the link labelled 'Submissions Needing Revision' to find your submission record. Your revised submission must include the following:

- a cover letter that should detail your responses to any editorial requests, if applicable, and whether changes have been made to the reference list

- a Response to Reviewers file that provides a detailed response to the reviewers' comments (if applicable, if not applicable please do not delete your existing 'Response to Reviewers' file.)

- a track-changes file indicating any changes that you have made to the manuscript.

NOTE: If Supporting Information files are included with your article, note that these are not copyedited and will be published as they are submitted. Please ensure that these files are legible and of high quality (at least 300 dpi) in an easily accessible file format. For this reason, please be aware that any references listed in an SI file will not be indexed. For more information, see our Supporting Information guidelines:

https://journals.plos.org/plosbiology/s/supporting-information

*Published Peer Review History*

Please note that you may have the opportunity to make the peer review history publicly available. The record will include editor decision letters (with reviews) and your responses to reviewer comments. If eligible, we will contact you to opt in or out. Please see here for more details:

https://plos.org/published-peer-review-history/

*Press*

Should you, your institution's press office or the journal office choose to press release your paper, please ensure you have opted out of Early Article Posting on the submission form. We ask that you notify us as soon as possible if you or your institution is planning to press release the article.

*Protocols deposition*

To enhance the reproducibility of your results, we recommend that if applicable you deposit your laboratory protocols in protocols.io, where a protocol can be assigned its own identifier (DOI) such that it can be cited independently in the future. Additionally, PLOS ONE offers an option for publishing peer-reviewed Lab Protocol articles, which describe protocols hosted on protocols.io. Read more information on sharing protocols at https://plos.org/protocols?utm_medium=editorial-email&utm_source=authorletters&utm_campaign=protocols

Please do not hesitate to contact me should you have any questions.

Sincerely,

Roli

Roland Roberts, PhD

Senior Editor

rroberts@plos.org

PLOS Biology

------------------------------------------------------------------------

DATA POLICY:

You may be aware of the PLOS Data Policy, which requires that all data be made available without restriction: http://journals.plos.org/plosbiology/s/data-availability. For more information, please also see this editorial: http://dx.doi.org/10.1371/journal.pbio.1001797

Note that we do not require all raw data. Rather, we ask that all individual quantitative observations that underlie the data summarized in the figures and results of your paper be made available in one of the following forms:

1) Supplementary files (e.g., excel). Please ensure that all data files are uploaded as 'Supporting Information' and are invariably referred to (in the manuscript, figure legends, and the Description field when uploading your files) using the following format verbatim: S1 Data, S2 Data, etc. Multiple panels of a single or even several figures can be included as multiple sheets in one excel file that is saved using exactly the following convention: S1_Data.xlsx (using an underscore).

2) Deposition in a publicly available repository. Please also provide the accession code or a reviewer link so that we may view your data before publication.

Regardless of the method selected, please ensure that you provide the individual numerical values that underlie the summary data displayed in the following figure panels as they are essential for readers to assess your analysis and to reproduce it: Figs 1CDE, 2ABCDE, 3ABCDEF, S1AB, S2ABCD, S3AB, S4ABCDE, S5ABCDEFG, S6ABCDEFGHIJ, 7ABCDEF. NOTE: the numerical data provided should include all replicates AND the way in which the plotted mean and errors were derived (it should not present only the mean/average values).

IMPORTANT: Please also ensure that figure legends in your manuscript include information on where the underlying data can be found, and ensure your supplemental data file/s has a legend.

Please ensure that your Data Statement in the submission system accurately describes where your data can be found.

------------------------------------------------------------------------

CODE POLICY

Per journal policy, if you have generated any custom code during the course of this investigation, please make it available without restrictions. Please ensure that the code is sufficiently well documented and reusable, and that your Data Statement in the Editorial Manager submission system accurately describes where your code can be found. More information on our Code Policy, what and how to share can be found here: https://journals.plos.org/plosbiology/s/code-availability

Please note that we cannot accept sole deposition of code in GitHub, as this could be changed after publication. However, you can archive this version of your publicly available GitHub code to Zenodo. Once you do this, it will generate a DOI number, which you will need to provide in the Data Accessibility Statement (you are welcome to also provide the GitHub access information). See the process for doing this here: https://docs.github.com/en/repositories/archiving-a-github-repository/referencing-and-citing-content

------------------------------------------------------------------------

DATA NOT SHOWN?

- Please note that per journal policy, we do not allow the mention of "data not shown", "personal communication", "manuscript in preparation" or other references to data that is not publicly available or contained within this manuscript. Please either remove mention of these data or provide figures presenting the results and the data underlying the figure(s).

------------------------------------------------------------------------

# Author comment: Author response to Decision Letter 4

**DOI:** 10.1371/journal.pbio.3003798.r007

27 Apr 2026

Attachment Submitted filename: Response_reviewers_R2_auresp_4.docx

# Editor report: Decision Letter 4

**DOI:** 10.1371/journal.pbio.3003798.r008

**Authors:** Roland Roberts

28 Apr 2026

Dear Theophane,

Thank you for the submission of your revised Discovery Report "Animal acoustic communication has a conserved optimal rhythm within the neural delta range" for publication in PLOS Biology. On behalf of my colleagues and the Academic Editor, Gail Patricelli, I'm pleased to say that we can in principle accept your manuscript for publication, provided you address any remaining formatting and reporting issues. These will be detailed in an email you should receive within 2-3 business days from our colleagues in the journal operations team; no action is required from you until then. Please note that we will not be able to formally accept your manuscript and schedule it for publication until you have completed any requested changes.

Please take a minute to log into Editorial Manager at http://www.editorialmanager.com/pbiology/, click the "Update My Information" link at the top of the page, and update your user information to ensure an efficient production process.

PRESS: We frequently collaborate with press offices. If your institution or institutions have a press office, please notify them about your upcoming paper at this point, to enable them to help maximise its impact. If the press office is planning to promote your findings, we would be grateful if they could coordinate with biologypress@plos.org. If you have previously opted in to the early version process, we ask that you notify us immediately of any press plans so that we may opt out on your behalf.

We also ask that you take this opportunity to read our Embargo Policy regarding the discussion, promotion and media coverage of work that is yet to be published by PLOS. As your manuscript is not yet published, it is bound by the conditions of our Embargo Policy. Please be aware that this policy is in place both to ensure that any press coverage of your article is fully substantiated and to provide a direct link between such coverage and the published work. For full details of our Embargo Policy, please visit http://www.plos.org/about/media-inquiries/embargo-policy/.

Thank you again for choosing PLOS Biology for publication and supporting Open Access publishing. We look forward to publishing your study.

Sincerely,

Roli

Roland G Roberts, PhD, PhD

Senior Editor

PLOS Biology

rroberts@plos.org
